# Traumatic Brain Injury Biomarkers, Simulations and Kinetics

**DOI:** 10.3390/bioengineering9110612

**Published:** 2022-10-25

**Authors:** Celeste Hicks, Akshima Dhiman, Chauntel Barrymore, Tarun Goswami

**Affiliations:** 1 Biomedical, Industrial and Human Factors Engineering, Wright State University, 3640 Col. Glen Hwy, Dayton, OH 45435, USA; 2 Boonshoft School of Medicine, Wright State University, 3640 Col. Glen Hwy, Dayton, OH 45435, USA

**Keywords:** TBI, PTSD, CTE, GFAP, UCH-L1, NF-L, total tau, S100B, BBB, kinetics, half-life

## Abstract

This paper reviews the predictive capabilities of blood-based biomarkers to quantify traumatic brain injury (TBI). Biomarkers for concussive conditions also known as mild, to moderate and severe TBI identified along with post-traumatic stress disorder (PTSD) and chronic traumatic encephalopathy (CTE) that occur due to repeated blows to the head during one’s lifetime. Since the pathways of these biomarkers into the blood are not fully understood whether there is disruption in the blood–brain barrier (BBB) and the time it takes after injury for the expression of the biomarkers to be able to predict the injury effectively, there is a need to understand the protein biomarker structure and other physical properties. The injury events in terms of brain and mechanics are a result of external force with or without the shrapnel, in the wake of a wave result in local tissue damage. Thus, these mechanisms express specific biomarkers kinetics of which reaches half-life within a few hours after injury to few days. Therefore, there is a need to determine the concentration levels that follow injury. Even though current diagnostics linking biomarkers with TBI severity are not fully developed, there is a need to quantify protein structures and their viability after injury. This research was conducted to fully understand the structures of 12 biomarkers by performing molecular dynamics simulations involving atomic movement and energies of forming hydrogen bonds. Molecular dynamics software, NAMD and VMD were used to determine and compare the approximate thermodynamic stabilities of the biomarkers and their bonding energies. Five biomarkers used clinically were S100B, GFAP, UCHL1, NF-L and tau, the kinetics obtained from literature show that the concentration values abruptly change with time after injury. For a given protein length, associated number of hydrogen bonds and bond energy describe a lower bound region where proteins self-dissolve and do not have long enough half-life to be detected in the fluids. However, above this lower bound, involving higher number of bonds and energy, we hypothesize that biomarkers will be viable to disrupt the BBB and stay longer to be modeled for kinetics for diagnosis and therefore may help in the discoveries of new biomarkers.

## 1. Introduction

Globally, an estimated 69 million people experience TBI [1] each year. Of the approximately 2.5 million annual cases of TBI in the United States alone, approximately 52,844 cases are fatal [2]. Motor vehicle accidents, sports injuries, falls [1], and blasts are some of the leading causes of TBI [2]. The high morbidity and mortality of TBI are partially due to limitations of current TBI diagnostic and prognostic methods [2]. Currently, neurological assessment tools, such as Glasgow Coma Scale (GCS), are used to rank injury severity, 13–15 representing mild or concussive injury and smaller numbers representing severe injury [2]. Neuroimaging techniques, namely positron emission tomography (PET), computed tomography (CT), and magnetic resonance imaging (MRI), are used to determine the extent of damage to brain tissue. However, neither neurological assessments nor neuroimaging techniques fully describe the patient’s prognosis for moderate to severe injury [2].

**Figure 1:**
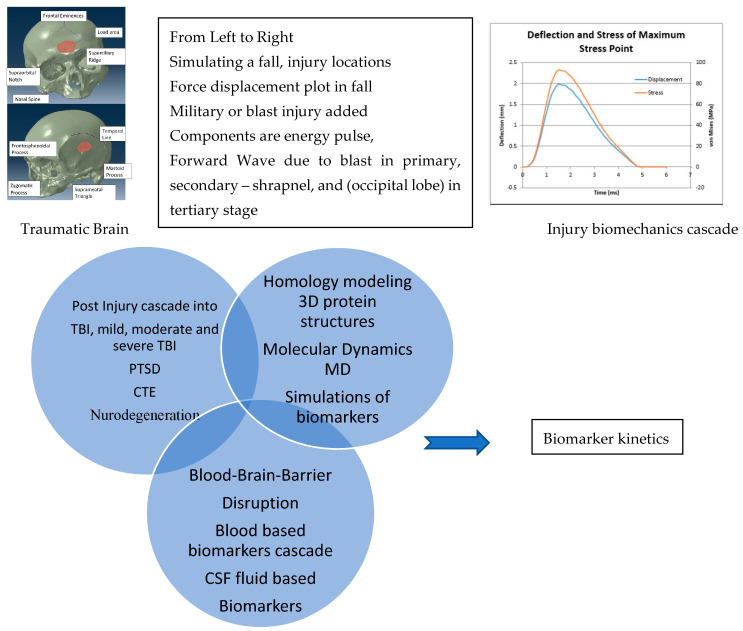
Injury from fall/blast with force, displacement and occipital injury, cascading into TBI, PTSD and CTE, investigated within protein homology, MD, blood–brain barrier and kinetics [1].

The cascade from the onset of injury causing TBI and to degenerative conditions, PTSD, has not been fully understood. Figure 1 shows injury locations resulting from fall in concussion/mTBI, however, additional components added to simulate a military blast scenario involving forward wave propagation and energy, shrapnel and occipital lobe in primary, secondary and tertiary stages, respectively. Post injury cascade is in terms of biomarkers, disruption in blood–brain barrier, and their kinetics has not been fully understood. Therefore, a measurable fluid based TBI biomarker in conjunction with imaging modalities will help diagnosis and prognosis [3]. Additionally, TBI transitioning into PTSD within a year of injury [4] or sequentially resulting in neurodegeneration has been documented. Both CTE and PTSD can be debilitating, chronic conditions [4].

Therefore, the objectives of this research were manyfold; readers interested in a specific topic are directed to specific sections at the end of each objective accomplished during this research. The objectives were to (1) identify TBI biomarkers from literature and perform homology modeling of their 3D protein structures (Section 2), (2) identify TBI biomarkers from clinical trials and their potential applications in diagnosis (Section 3), (3) perform Molecular Dynamics (MD) simulations of biomarkers (Section 4), (4) blood–brain barrier cascades and metabolic pathways of select biomarkers (Section 5) and (5) biomarker kinetics so that diagnostic capabilities improved for prognosis and rehabilitation (Section 6). This paper discusses each of these objectives in this sequence (1–5). The temporal profiles of these TBI biomarkers and the mechanisms by which the biomarkers become overexpressed and pass through the blood–brain barrier into the blood stream were reviewed. We identified biomarkers from literature for TBI/CTE/PTSD in Table 1.

## 2. Biomarkers

**Figure 2:**
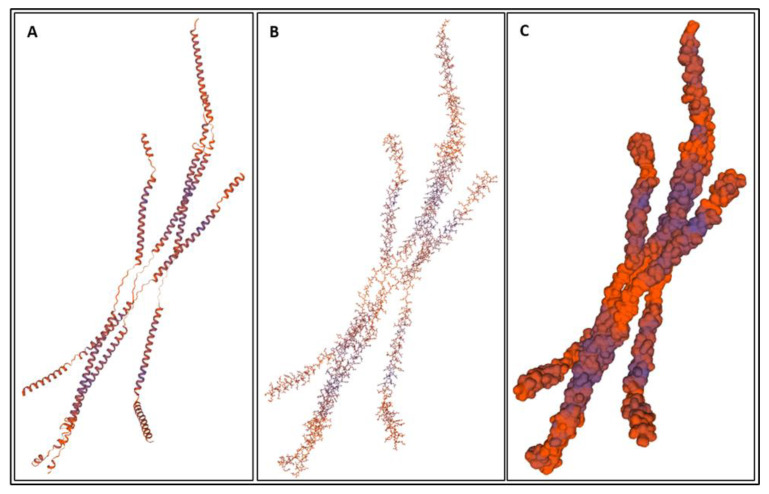
(**A**) A cartoon model, (**B**) a ball-and-stick model, and (**C**) a surface model of GFAP [24].

**Figure 3:**
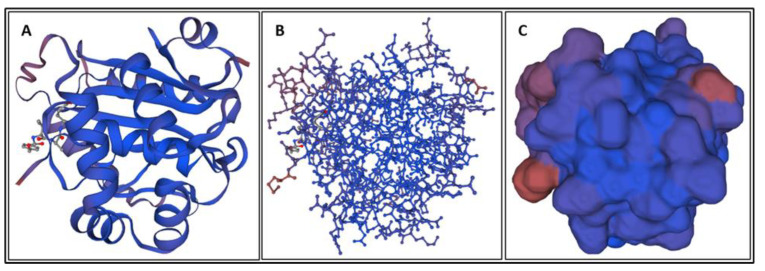
(**A**) A cartoon model, (**B**) a ball-and-stick model, and (**C**) a surface model of UCH-L1 [26].

**Figure 4:**
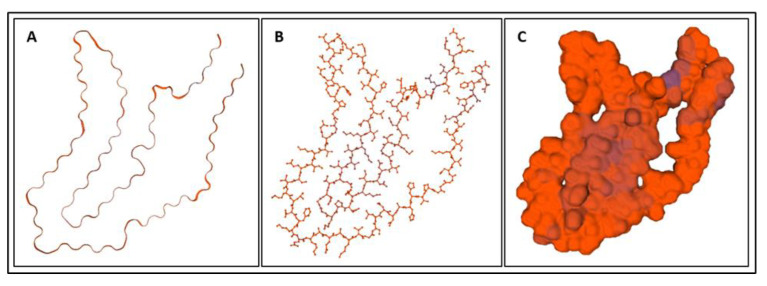
(**A**) A cartoon model, (**B**) a ball-and-stick model, and (**C**) a surface model of MAP-2 [28].

**Figure 5:**
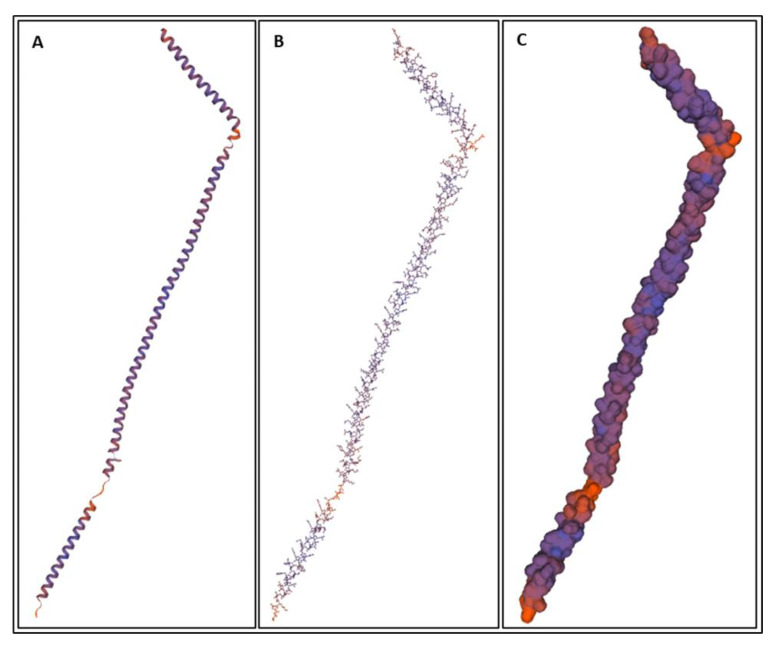
(**A**) A cartoon model, (**B**) a ball-and-stick model, and (**C**) a surface model of NF-L [30].

**Figure 6:**
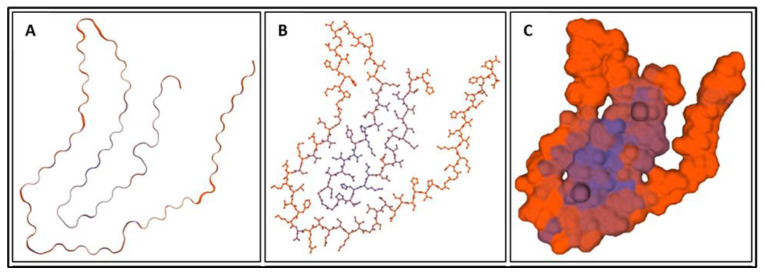
(**A**) A cartoon model, (**B**) a ball-and-stick model, and (**C**) a surface model of tau isoform 0N3R (tau-352) [32].

**Figure 7:**
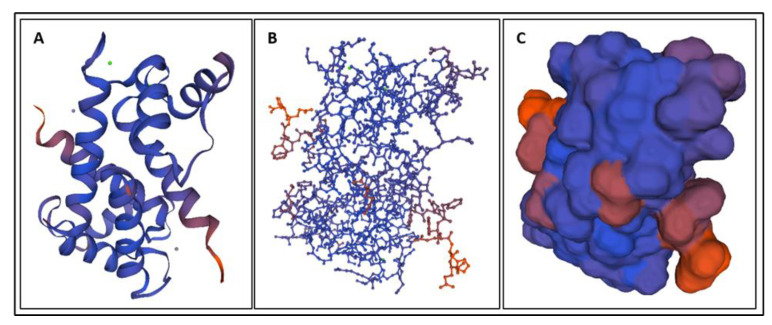
(**A**) A cartoon model, (**B**) a ball-and-stick model, and (**C**) a surface model of S100B [34].

**Figure 8:**
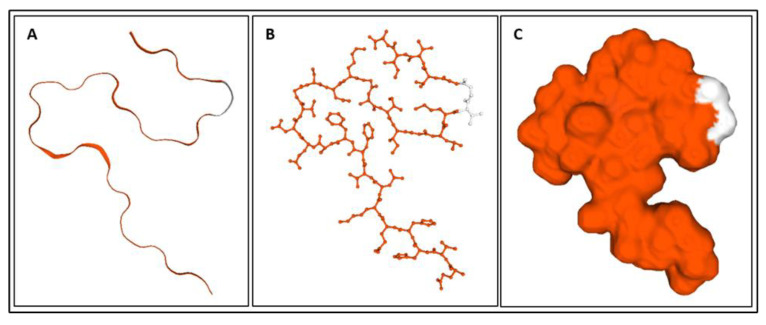
(**A**) A cartoon model, (**B**) a ball-and-stick model, and (**C**) a surface model of Aβ42 made using amino acid sequences from the GenScript website [36].

**Figure 9:**
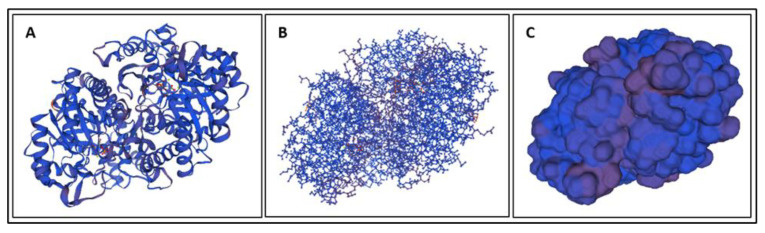
(**A**) A cartoon model, (**B**) a ball-and-stick model, and (**C**) a surface model of NSE made using amino acid sequences from the UniProt database [38].

**Figure 10:**
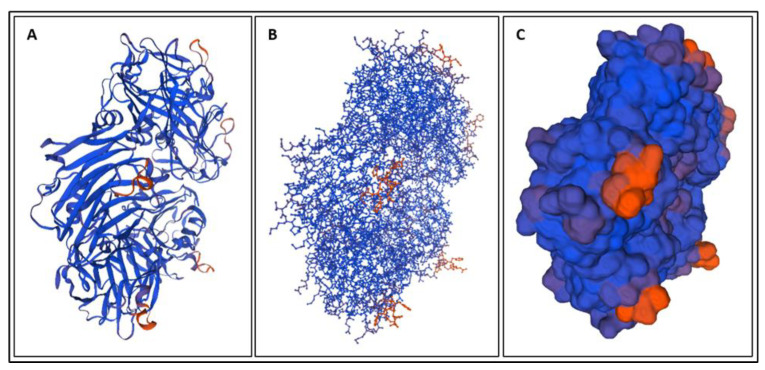
(**A**) A cartoon model, (**B**) a ball-and-stick model, and (**C**) a surface model of CRP made using amino acid sequences from the UniProt database [40].

**Figure 11:**
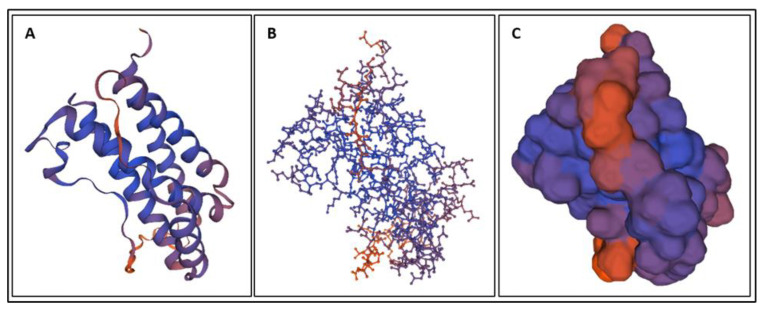
(**A**) A cartoon model, (**B**) a ball-and-stick model, and (**C**) a surface model of IL-6 made using amino acid sequences from the UniProt database [42].

**Figure 12:**
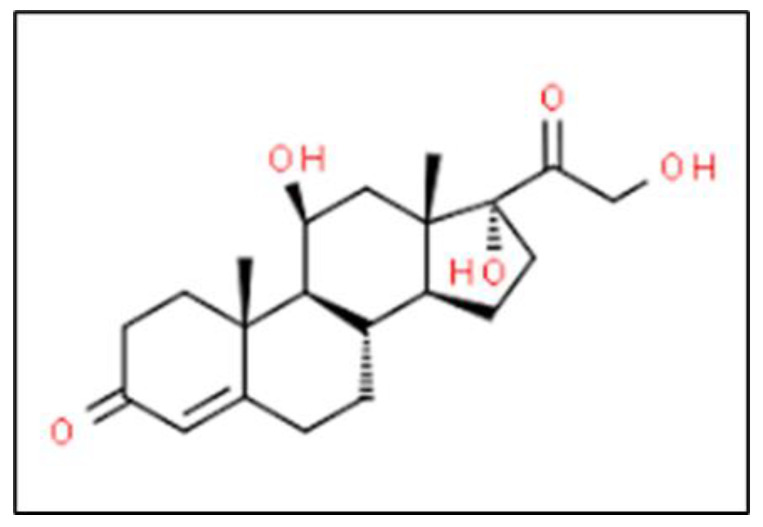
The chemical structure of cortisol [43].

**Figure 13:**
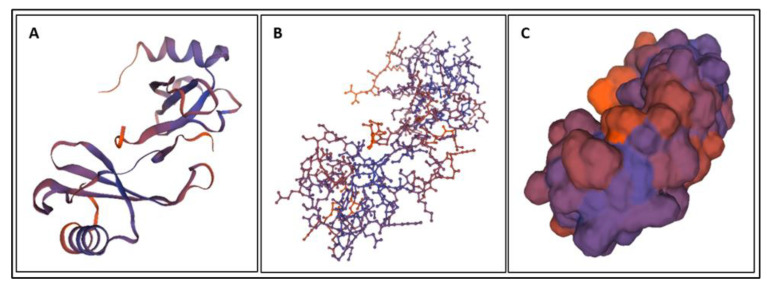
(**A**) A cartoon model, (**B**) a ball-and-stick model, and (**C**) a surface model of CCL11 made using amino acid sequences from the UniProt database [45].

Biomarkers were identified from literature. Homology modeling of 3D protein structures was performed using SWISS MODEL, Figure 2, Figure 3, Figure 4, Figure 5, Figure 6, Figure 7, Figure 8, Figure 9, Figure 10, Figure 11, Figure 12 and Figure 13. All models generated by SWISS MODEL were unaltered and are licensed under the following copyright license: Available online: https://creativecommons.org/licenses/by-sa/4.0/legalcode (accessed on 10 June 2022). The amino acid sequence was obtained from one of the following databases: NCBI protein database, UniProt, GenScript [18,19,20,21,22,23,24,25,26,27,28,29,30,31,32,33,34,35,36,37,38,39,40,41,42,43,44,45,46,47,48,49,50,51,52,53,54,55,56,57,58,59,60,61,62,63,64,65,66,67,68,69].

### 2.1. GFAP

Glial fibrillary acidic protein (GFAP) is an intermediate filament protein predominately expressed in the central nervous system [23]. GFAP measured in the blood is an indicator of TBI, together with abnormal CT scan results right after injury, and poor long-term physical outcome after TBI.

### 2.2. UCH-L1

Ubiquitin carboxy-terminal hydrolase L1 (UCH-L1) is a deubiquitinase involved in the metabolism removal of other proteins from the cell [25]. Blood concentration of UCH-L1 serves as an indicator of TBI, together with abnormal CT following injury, and poor long-term neurological outcome after TBI.

### 2.3. MAP-2

Microtubule-associated protein-2 (MAP-2) is a neuron-specific protein that acts to stabilize microtubules [27]. MAP-2 measured in cerebrospinal fluid has limited predictive ability, abnormal head CT results right after TBI injury and good predictive ability of early mortality from TBI.

### 2.4. NF-L

Neurofilament light (NF-L) is a subunit of neurofilament proteins, which are one of the most abundant proteins in the brain and neuron-specific [29]. Blood NF-L concentration being a good predictor of TBI diagnosis along with abnormal head CT results following TBI.

### 2.5. T-Tau

Total tau (t-tau) is a microtubule-associated protein found in the brain [31]. Limited evidence of blood t-tau concentration being a decent predictor of TBI and abnormal CT findings following TBI.

### 2.6. S100B

S100B is a calcium-binding protein largely expressed in astrocytes and is involved in the regulation of brain cells’ energy metabolism [33]. S100B blood concentration was not indicative of TBI and abnormal CT findings after injury and was largely affected by other types of tissue damage besides the TBI.

### 2.7. Aβ42

Amyloid beta peptide 42 (Aβ42) is a peptide that is closely associated with neuroinflammation, neurodegeneration, and cognitive impairment in other neurological diseases, namely Alzheimer’s Disease [35]. We found evidence of blood Aβ42 concentration as an indicator of mTBI but not being related to the post-concussive or behavioral symptoms of TBI.

### 2.8. NSE

Neuron-specific enolase (NSE) is a glycolytic enzyme associated with neural degeneration, inflammation, and regeneration [37]. Blood NSE was an indicator of TBI but was not directly correlated with TBI severity.

### 2.9. CRP

C-reactive protein (CRP) a member of the pentraxin superfamily of protein and is involved with the regulation of the human immune system, with one specific function being triggering inflammation [39]. Blood CRP concentration was affected by multiple types of tissue damage, not just brain damage, and a predictor of 6-month neurological outcome after TBI.

### 2.10. IL-6

Interleukin-6 (IL-6) is a cytokine involved with regulating the immune system, controlling body weight and glucose tolerance and influencing various behavioral traits [41]. IL-6 concentration in the blood is correlated with repetitive TBIs and with PTSD symptoms in people with a history of repetitive TBIs.

### 2.11. Cortisol

Cortisol is a hormone involved in the stress response. We found limited evidence of blood cortisol levels being associated with a history of emotional trauma that had the potential to cause PTSD.

### 2.12. CCL11

Eotaxin (CCL11) is a chemokine involved in the recruitment of eosinophils to inflammatory sites during allergic reactions and is associated with neurogenesis and neurodegeneration [44]. Limited evidence of cerebrospinal fluid CCL11 concentration being able to differentiate between subjects with and without CTE.

Additionally, physical properties of these biomarkers are shown in Table 2.

The amino acid lengths and isoelectric points of the biomarkers in Table 2 were checked in MATLAB for a potential correlation. A very weak correlation coefficient of −0.264, indicating no significant difference among them to be able to predict preference of one biomarker over other. The basal isoelectric point is important in many methodologies for separating and measuring proteins from biological samples, such as electrophoresis. Isoelectric specifically has been shown to be able to separate proteins with pI differences of as little as 0.01 [70]. The biomarkers listed in Table 2 all had pI differences of at least this value; therefore, protein concentration and measurement methods such as electrophoresis may be viable for the separation and detection of these biomarkers individually or simultaneously.

Even though the molecular weight of biomarkers is shown in table above, in terms of kg/mol, they can be classified within low, medium and high categories. Since S100B, GFAP, UCH-L1, NF-L and t-tau were found to be more applicable to connect clinically TBI conditions these proteins showed great potential to be clinically useful. These biomarkers showed strong predictive abilities and kinetics for TBI conditions and their symptoms. These biomarkers are discussed further:

Glial fibrillary acidic protein (GFAP) is an intermediate filament protein predominately expressed by astrocytes in the central nervous system [23,67]. GFAP plays a key role in astrocytic processes that aid in the regulation of neuron synapses [68]. Studies have also shown that GFAP plays an important role in the regeneration of neuronal axons and in the regulation of inflammation [67]. Production of GFAP is stimulated by damage to the astrocyte [23,68,69]. As such, upregulation of GFAP is indicative of central nervous system repair following cell injury or death [68]. Disintegration of the astrocyte skeleton causes GFAP to be released into the blood stream [23,69]. The presence of GFAP in the blood has been observed following brain injury, stroke, and other neurodegenerative disorders [69]. The high specificity of GFAP to the central nervous system and the observance of GFAP in the blood following TBI have encouraged research into the viability of using GFAP as a biomarker of TBI [23,67,68,69].

Ubiquitin carboxy-terminal hydrolase L1 (UCH-L1) is a deubiquitinase involved in the addition and removal of ubiquitin from other proteins during their metabolism [25,65]. Additionally, UCH-L1 plays a key role in the removal of unwanted proteins from the cell [25]. UCH-L1 has been associated with the progression of other neurological conditions including Alzheimer’s Disease and Parkinson’s Disease [65]. UCH-L1 is another of the most abundant proteins in the brain, making up-to approximately 5% of the total protein in neurons [65,66]. Although some UCH-L1 is found in cells outside of the nervous system, the vast majority of UCH-L1 is expressed in the brain, and it is highly neuron-specific [25,66]. The abundance of UCH-L1 in neurons along with the high specificity of UCH-L1 to the brain has inspired the idea of using UCH-L1 as a biomarker for TBI [66].

Neurofilament light (NF-L) is one of four subunits that compose neurofilament proteins [58]. Neurofilament proteins are neuron-specific and one of the most abundant proteins in the brain [29,58]. Together with microtubules and actin filaments, neurofilament proteins compose the neuronal cytoskeleton [59]. The neuronal cytoskeleton provides the structure of the neuron and is essential for the axon’s specialized structures and functions [59]. NF-L specifically is essential for the proper growth, nerve conduction, and regeneration of mature axons [60]. Neurodegeneration and neuronal injury strongly impact neurofilament proteins and cause them to be released into the blood and cerebrospinal fluid at large [29]. NF-L specificity to the brain, relation with neuron regeneration, sensitivity to neuronal injury, and susceptibility to being released into the blood have sparked interest into using NF-L as a biomarker for TBI [29].

Tau is a neuronal microtubule-associated protein [31]. As a microtubule-associated protein, tau largely works to regulate the actions of and stabilize microtubules, especially the microtubules in the elongated neuronal axons of the brain [31,61,62]. There are six major isoforms of tau plus phosphorylated forms of each isoform: total tau (t-tau) is the measure of all phosphorylated and non-phosphorylated tau isoforms together [61]. Elevations in the concentration of tau are indicative of axonal injury and have been observed in response to concussion and other head trauma [62,63]. Additionally, accumulations of tau in the brain are associated with other neurodegenerative diseases, including Alzheimer’s Disease and chronic traumatic encephalopathy [62,63,64]. T-tau, phosphorylated-tau (p-tau), and the ratio of p-tau to t-tau have all been investigated as potential biomarkers of TBI, but this review focuses on t-tau.

## 3. Biomarkers from Clinical Trials

Biomarkers presented for TBI, CTE, and PTSD from clinical trials are summarized in Table 3. This effort will establish biomarkers from literature applied in clinics.

### 3.1. Clinical Evaluation

Clinical evaluation of TBI, PTSD and CTE require diagnostic tools, biomarkers, and imaging modalities so that prognosis understood and treatment and/or rehabilitation methods prescribed or implemented. In scenarios where either CT or MRI is inconclusive, biomarkers may be useful. However, too long after the injury the biomarkers deplete, therefore, tests are to be conducted either in conjunction with imaging or soon thereafter. The section below discusses several biomarkers for their ability to detect TBI with diagnostics, repetitive TBI (for CTE), imaging and functional outcomes derived from [62,63,68,80,81,82,83,84,85,86,87,88,89]. A summary of clinical trial studies with reference, number of subjects, inclusion, and exclusion criteria, TBI severities, time since last injury and biomarkers investigated was summarized in Appendix A.

#### 3.1.1. NF-L TBI Diagnosis

Ability to differentiate between patients with and without TBI significantly decreases or is insignificant by approximately one year post injury [62,80,81]. However, NF-L has been shown to have excellent predictive ability of TBI when measured between approximately 2-weeks and 6 months post injury [82,83]. Its ability to predict moderate-to-severe TBI also appears to be stronger than its ability to predict mild TBI [82,83].

Repetitive TBI: increased NF-L concentrations. It has been correlated with the number of TBI injuries suffered [62,84]. NF-L was shown to be significantly elevated in people with a history of repetitive TBIs 3 or more lifetime TBIs compared to controls with no TBI history, but the difference in NF-L concentrations was the most significant when comparing people with a history of 1–2 TBIs to people with a history of repetitive TBIs [62,84].

Head Scan Findings: NF-L concentration shortly after injury has been correlated with abnormal findings on head CT and MRI scans [85,86]. NF-L has been shown to have excellent ability to distinguish between patients with and without abnormal head scan results [85]. Additionally, NF-L concentration has been shown to be significantly elevated in patients with either isolated contusion only or isolated DAI only compared to controls, as well as in patients with diffuse injury compared to those with focal injury [85,86].

Functional Outcome: NF-L appears to be correlated with both GOS-E score and with changes in GOS-E score [80,82]. Increased NF-L concentration has been associated with a worse coincident GOS-E score both shortly after injury and at 1 year post injury [80,82]. However, NF-L concentration measured at 30 days post injury has been associated with an improvement in GOS-E score by 90 days post injury [82].

#### 3.1.2. T-Tau TBI Diagnosis

Tau concentration has been shown to have a weak to moderate ability to distinguish between people with and without a TBI or a history of TBI [62,63,81,82,83]. Tau appears to be a moderate indicator of mild TBI when measured acutely after injury; however, it does not appear to be significantly useful in identifying TBI in the non-acute phases of injury [62,63,81,82,83].

Repetitive TBI: limited evidence exists that correlated tau concentration with the number of TBIs suffered in the course of lifetime when measured within approximately 3 years of injury [63]. Tau concentration measured after approximately 3 years post injury, however, did not correlate with repetitive TBIs [62]. Given the apparent variability in tau’s concentration over time, it is plausible that tau may be correlated with repetitive TBI for only a limited amount of time after injury.

Head Scan Findings: Tau has been shown to have moderate predictive ability for abnormal head CT or MRI scan results when measured in the acute phase after injury [83,85,86,87]. However, tau does not appear to be indicative of the specific type of injury seen in the scan [85,86].

Functional Outcome: Tau concentration does not appear to be significantly associated with GOS-E score, changes in GOS-E score, or the presence or absence of mild neurocognitive disorder; however, evidence was only limited [81,82].

#### 3.1.3. UCH-L1 TBI Diagnosis

UCH-L1 has been shown to have poor ability to discriminate between patients with mild-to-moderate TBI and those without TBI for up to 5 years post injury [82,88]. However, UCH-L1 has been shown to have a moderate ability to differentiate between patients with moderate-to-severe TBI and those without TBI when measured at approximately 1 month post injury [82]. Despite this difference, however, UCH-L1 does not appear to be correlated with Glasgow Coma Scale score [86].

Head Scan Findings: UCH-L1 appears to be a good predictor of abnormal CT scan results only when the presence of TBI is unaccounted for [68,85,88,89]. UCH-L1 has been shown to have excellent predictive ability for abnormal CT scan results among head trauma patients with and without a diagnosed TBI [85,88,89]. However, UCH-L1 has been shown to perform poorly when used to discriminate between normal and abnormal CT scan results among TBI patients only [68]. Additionally, UCH-L1 appears to not be correlated with the type of injury seen on the CT scan [86]. This all suggests that UCH-L1 may not be a useful predictor of CT scan results in patients with confirmed TBIs.

Functional Outcome: UCH-L1 does not appear to be correlated with GOS-E score or changes in GOS-E score [82,89].

#### 3.1.4. GFAP TBI Diagnosis

GFAP appears to be a moderate to excellent indicator of moderate-to-severe TBI for up to 5 years post injury [80,81,82]. GFAP also appears to be a moderate to excellent indicator of mild TBI when measured within several days to weeks of injury [80,82,83,88]. Additionally, GFAP appears to have moderate to excellent ability to discriminate between severe TBI and mild-to-moderate TBI; however, this ability may not be present until several days or months after injury [80,81,82].

Head Scan Findings: GFAP has been shown to be an excellent predictor of abnormal CT or MRI findings [68,83,85,86,87,88,89]. GFAP has been shown to have excellent ability to discriminate between patients with and without abnormal head scan results for at least 7 days after injury, with the best discriminative ability within a few days of injury [68,83,85,86,87,88,89]. This ability is also maintained when GFAP is used to predict abnormal head scan results only among patients with a diagnosed TBI [68]. Some evidence indicates that GFAP’s predictive ability for abnormal head scan results is dependent on age, however, with GFAP having significantly lower predictive ability for CT scan results in people aged 60 years or older [87].

Functional Outcome: GFAP may have a low correlation with concurrent and future GOS-E scores [80,81,82,89]. There is evidence that GFAP concentration is loosely associated with future GOS-E scores and with changes in GOS-E scores [80,81,82,89]. However, the evidence for this is limited, and these associations only appear to be present at certain time points post injury [80,81,82,89].

All four biomarkers demonstrated some level of ability to indicate the presence of TBI. NF-L was best used for identifying moderate-to-severe TBI within approximately 2 weeks to 6 months post-injury; tau was best used for identifying mild TBI acutely after injury; UCH-L1 was best used for identifying moderate-to-severe TBI at 1 month post-injury; and GFAP was capable of identifying moderate-to-severe TBI for up to 5 years and mild TBI for up to a few weeks post-injury. Overall, GFAP demonstrated the strongest ability to identify TBI by being capable of identifying any severity of TBI for the longest amount of time. However, since NF-L and UCH-L1 can only indicate moderate-to-severe TBI, tau can only indicate mild TBI, and GFAP can indicate all severities, NF-L, tau, and UCH-L1 may also be useful for confirming GFAP’s diagnosis of TBI severity.

NF-L and tau both demonstrated some correlation with repetitive TBI. NF-L, however, was more capable predicting repeat TBI than tau. For this reason, NF-L appears to be the preferred indicator of repetitive TBI of the four biomarkers discussed in this paper.

All four biomarkers also demonstrated some level of ability to predict abnormal head scan results shortly after injury. Of the four, GFAP demonstrated the strongest ability to indicate TBI for patients younger than 60 years old. For older than 60 years of age, head scan results were more preferred than GFAP biomarker. However, given NF-L and head scan were able to predict injury type which was not the case with UCH-L1. NF-L may be the most appropriate indicator of TBI injury suffered.

Tau and UCH-L1 did not appear to be correlated with GOS-E score or changes in GOS-E score. GFAP demonstrated a low association with GOS-E, but this relationship did not appear to be stable or very reliable. NF-L was the only biomarker to be correlated with GOS-E score and appears to be capable of predicting concurrent GOS-E score as well as some improvement in GOS-E score when measured at 1 month post injury

## 4. Molecular Dynamics Simulations

In order to fully understand TBI biomarkers at the molecular level, their protein structures were modelled and simulated using the molecular dynamics software NAMD and VMD; version 2.14 Win64 and version 1.9.3 Windows OpenGL (32-bit Intel x86) (University of Illinois, IL USA). Eight of the ten TBI biomarkers were simulated: Aβ42, IL-6, CRP, S100B, NSE, GFAP, UCH-L1, and tau. The NAMD simulations required a .pdb file from the RCSB protein data bank (PDB) for each protein. NF-L and MAP-2 did not have entries in the RCSB PDB and therefore could not be simulated. Additionally, two different presentations of tau were simulated: straight filament (SF) tau and paired helical filament (PHF) tau. SF tau and PHF tau are the two ways that tau is deposited in neurons in various tauopathies, such as Alzheimer’s Disease. Both formations were modelled to illustrate and investigate the tauopathy aspect of TBI more completely.

The PDB entry selected to represent each biomarker was as follows: 6szf for Aβ42, 1alu for IL-6, 1gnh for CRP, 3d0y for S100B, 3ucc for NSE, 6a9p for GFAP, 2etl for UCH-L1, 7mkg for SF tau, and 7mkh for PHF tau. Each protein was simulated in a water box at 310° K for 5 ps. The data from these simulations were used to plot the bond, electrostatic, potential, kinetic, and total energies for each protein over the course of the simulation.

### 4.1. Biomarkers

**Figure 14:**
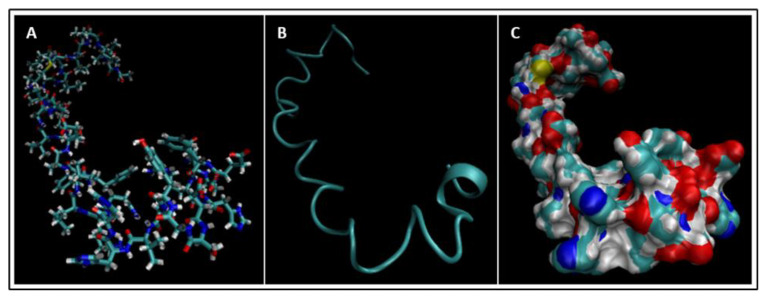
(**A**) A cartoon model, (**B**) a bonds model, and (**C**) a surface model of Aβ42.

**Figure 15:**
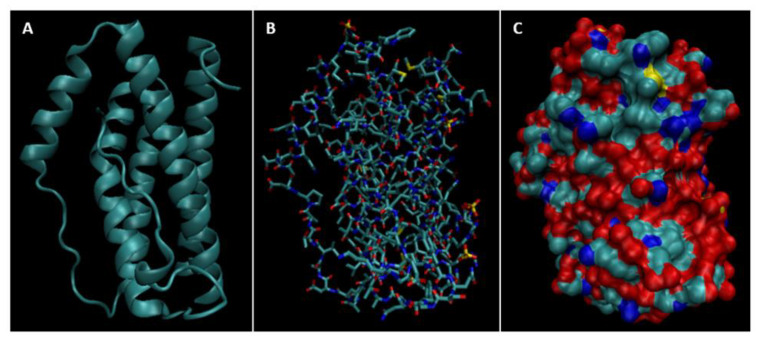
(**A**) A cartoon model, (**B**) a bonds model, and (**C**) a surface model of IL-6.

**Figure 16:**
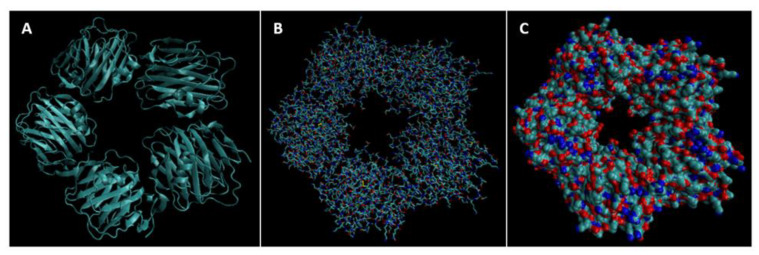
(**A**) A cartoon model, (**B**) a bonds model, and (**C**) a surface model of CRP.

**Figure 17:**
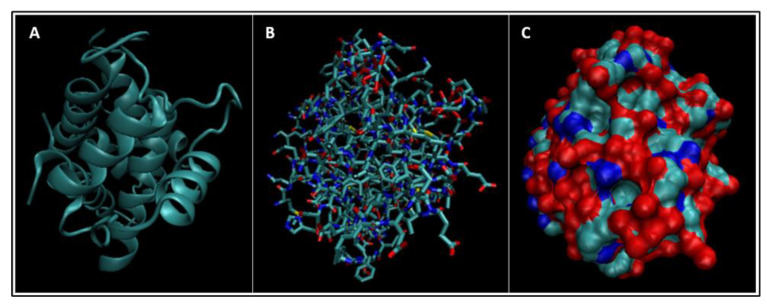
(**A**) A cartoon model, (**B**) a bonds model, and (**C**) a surface model of S100B.

**Figure 18:**
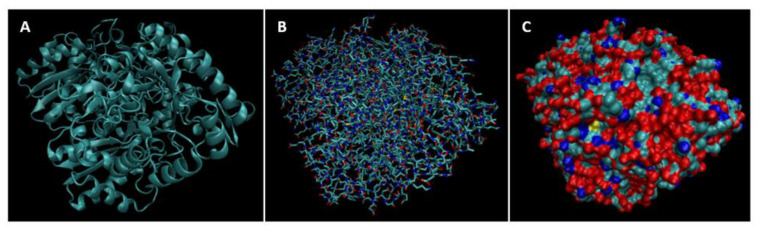
(**A**) A cartoon model, (**B**) a bonds model, and (**C**) a surface model of NSE.

**Figure 19:**
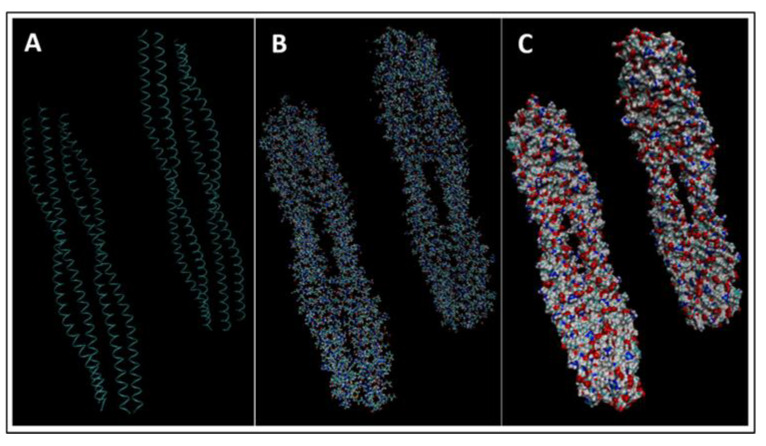
(**A**) A cartoon model, (**B**) a bonds model, and (**C**) a surface model of GFAP.

**Figure 20:**
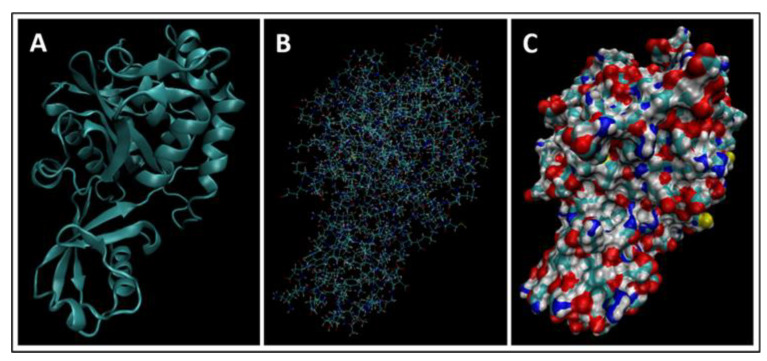
(**A**) A cartoon model, (**B**) a bonds model, and (**C**) a surface model of UCH-L1.

**Figure 21:**
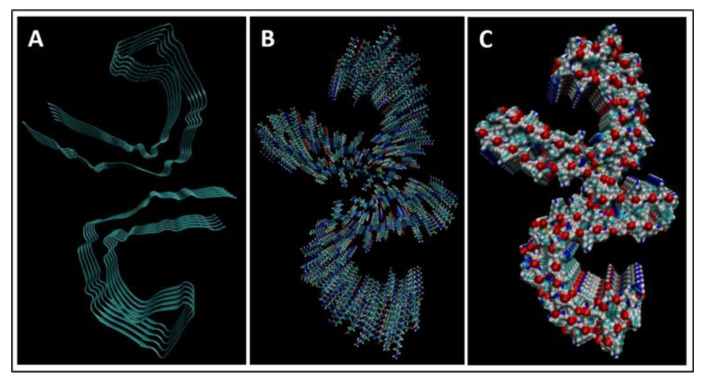
(**A**) A cartoon model, (**B**) a bonds model, and (**C**) a surface model of SF tau.

**Figure 22:**
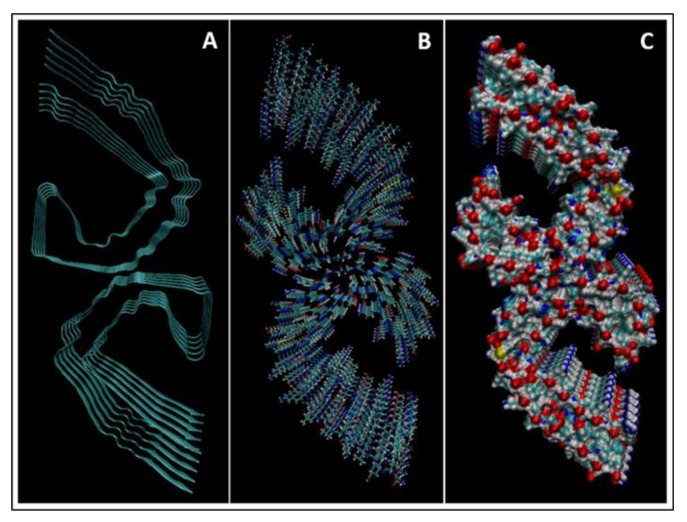
(**A**) A cartoon model, (**B**) a bonds model, and (**C**) a surface model of PHF tau.

The results of the simulations are presented below for each of the biomarkers. Figure 14, Figure 15, Figure 16, Figure 17, Figure 18, Figure 19, Figure 20, Figure 21 and Figure 22 show the biomarker models obtained from NAMD and VMD. The colors in the biomarker surface models correspond to atoms. The default VMD atom color code was used: white representing H atoms, red O, blue N, cyan C, yellow S, tan P, and silver Z atoms, respectively.

### 4.2. Molecular Energy and Orbital Diagrams

Simulations were run using the water boxes, Kit 3 VMD version 1.9.3, the number of water molecules in the box for each protein is as follows: GFAP: 57735, UCH-L1: 12154 SF Tau: 14550 PHF Tau: 14678, IL6:4465, NSE: 15967, S100B: 4335, Ab42: 2441, CRP: 72375. Biomarkers have bonding capabilities. As bonds form, the electrons in those bonds are paired in molecular orbitals. When energy is absorbed by molecules, bonded electrons use that energy to be temporarily promoted from the highest occupied molecular orbital (HOMO) to the lowest unoccupied molecular orbital (LUMO). This movement is known as electron excitation and is depicted by energy spectra (see below). During this transition, the original unexcited state of the electron is called the ground-state singlet state (S_0_) and the first excited state of the molecule is called a singlet state (S_1_).

**Figure 23:**
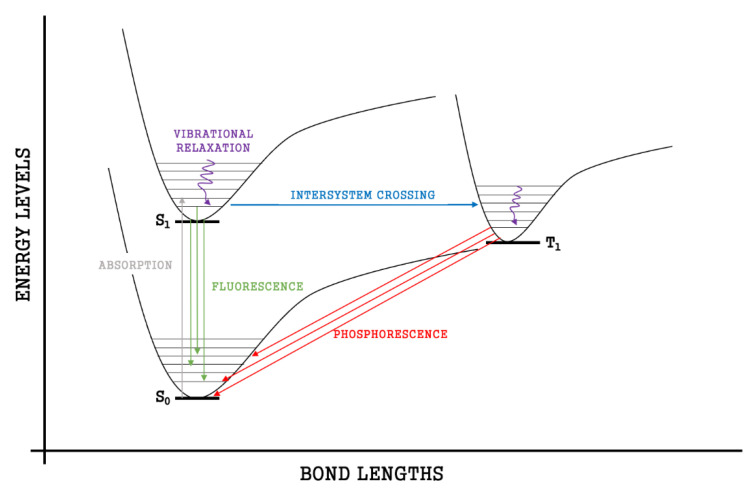
Difference in energy states show how the S_0_, S_1_, and T_1_ states create the molecular dynamic curves.

**Figure 24:**
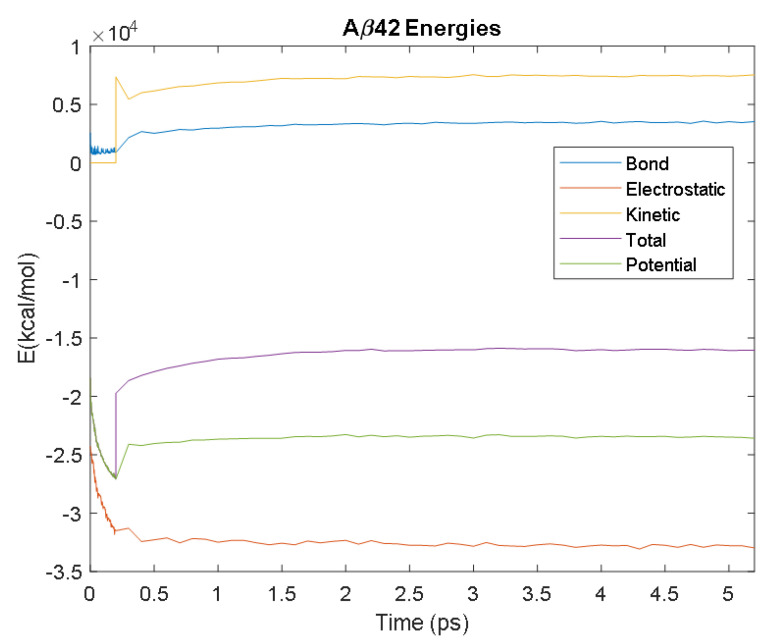
A plot of Aβ42′s bond, electrostatic, kinetic, total, and potential energies over 5 ps from its simulation in NAMD.

**Figure 25:**
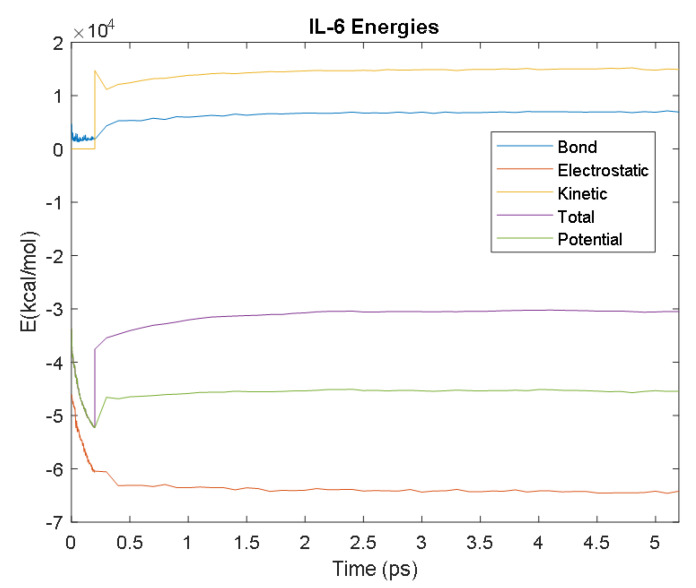
A plot of IL-6′s bond, electrostatic, kinetic, total, and potential energies over 5 ps from its simulation in NAMD.

**Figure 26:**
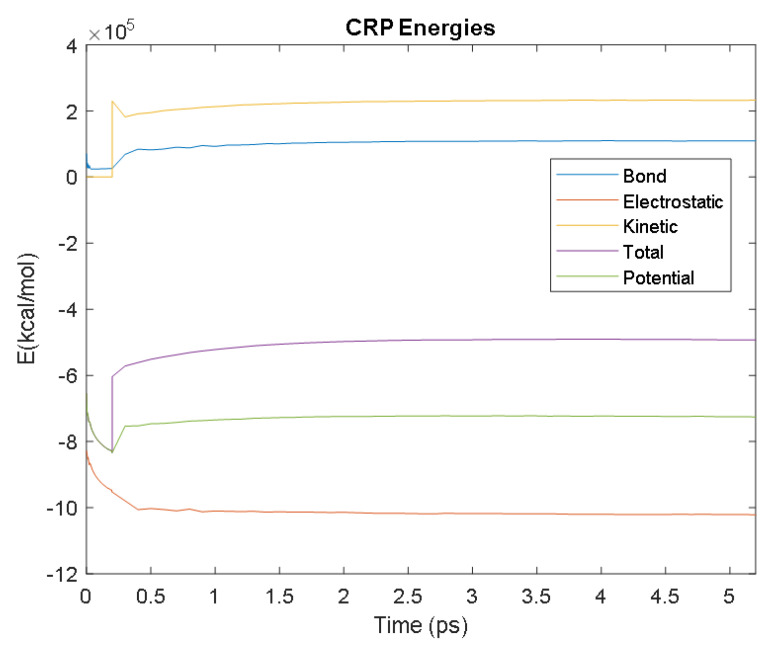
A plot of CRP’s bond, electrostatic, kinetic, total, and potential energies.

**Figure 27:**
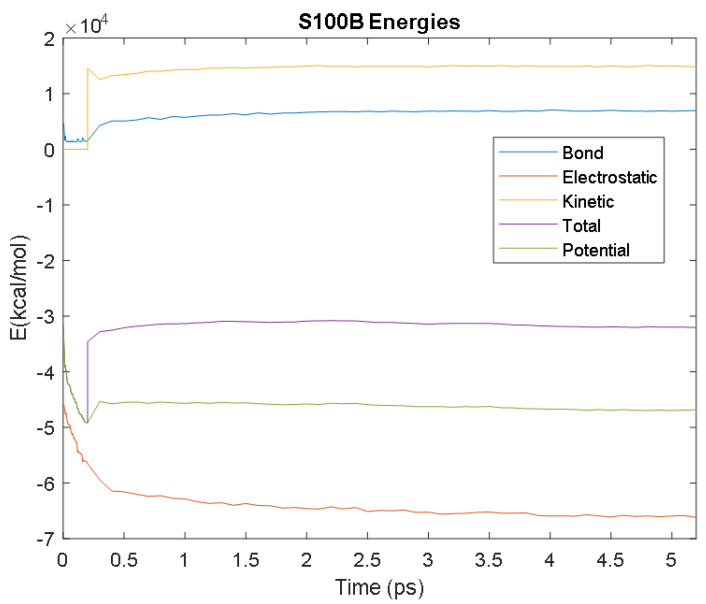
A plot of S100B’s bond, electrostatic, kinetic, total, and potential energies.

**Figure 28:**
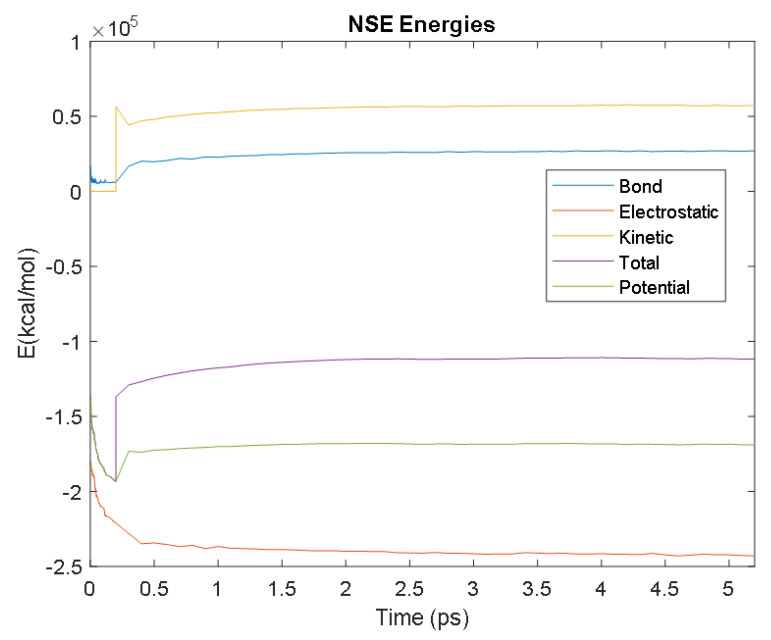
A plot of NSE’s bond, electrostatic, kinetic, total, and potential energies.

**Figure 29:**
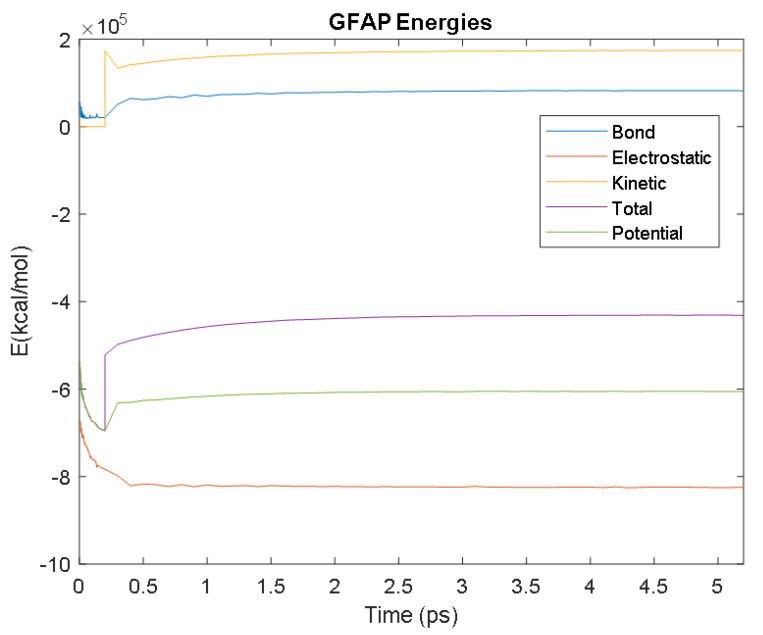
A plot of GFAP’s bond, electrostatic, kinetic, total, and potential energies.

**Figure 30:**
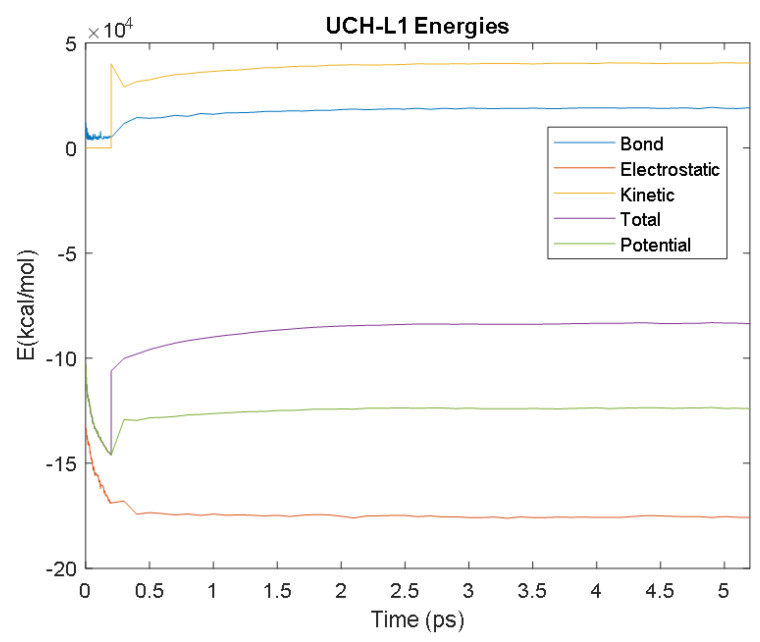
A plot of UCH-L1′s bond, electrostatic, kinetic, total, and potential energies.

**Figure 31:**
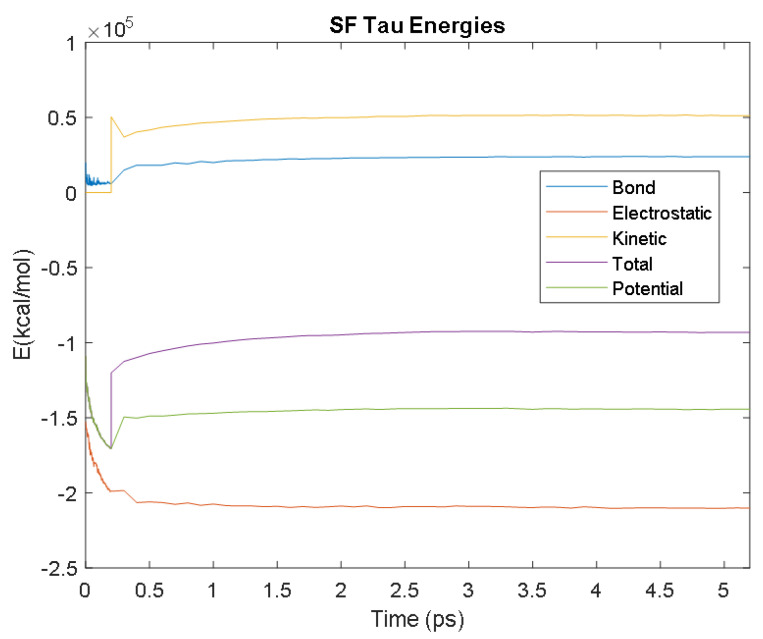
A plot of SF tau’s bond, electrostatic, kinetic, total, and potential energies.

**Figure 32:**
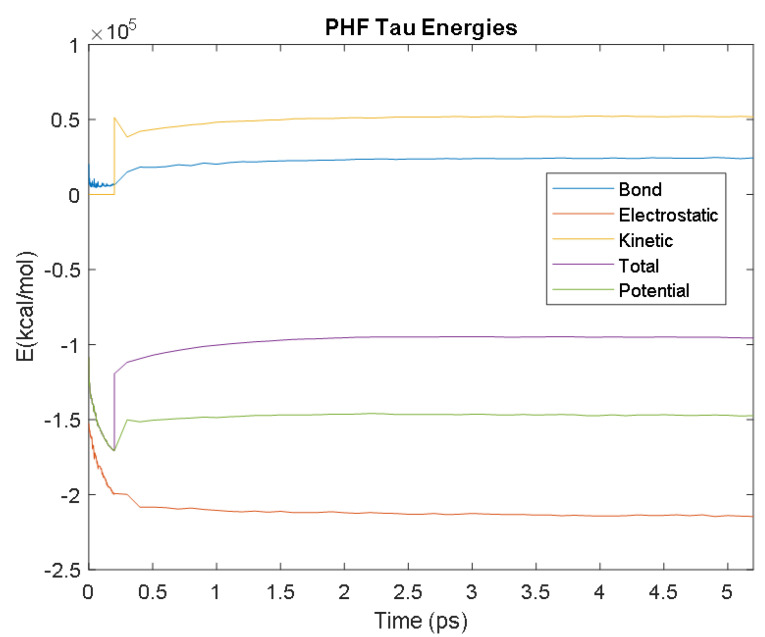
A plot of PHF tau’s bond, electrostatic, kinetic, total, and potential energies.

Every electron has a fixed amount of angular momentum associated with it and this intrinsic value is known as spin (either spin up or down). As the electron jumps from S_0_ to S_1_, it retains its spin direction. Electrons reverting to their ground state will re-emit the energy they absorbed but delayed in time and at a different energy than the absorbed energy. Fluorescence, the quicker of the two pathways, is simply the return of an electron from S_1_ to its ground-state. Alternatively, the excited electron (S_1_) may undergo a change in spin through intersystem crossing (a radiation-less transfer between states) to enter the triplet state, T_1_. This change in spin helps stabilize the excited electron, which is why the T_1_ state is at a lower energy level than the S_1_ state, but still at a higher energy level than the S_0_ state. From the T_1_ state, Figure 23, the excited electron can relax to its ground state via phosphorescence, the emission of energy from an electron in a different state than which it was initially excited into. This trio of energy states for any given electron in any bond of our biomarkers creates the distinct curve shown by some of the energy curves in the biomarker’s energy plots. These energy plots were created in MATLAB using data from NAMD and are shown in Figure 24, Figure 25, Figure 26, Figure 27, Figure 28, Figure 29, Figure 30, Figure 31 and Figure 32. We presented energy in kcal/mol on the *Y*-axis and time in pico seconds is on the horizontal axis.

It was observed from the energy plots that all energies for each biomarker was able to reach thermodynamic equilibrium. MATLAB was then used to determine the approximate time to reach equilibrium for each energy and the average value of each simulation presented in Table 4.

The energy data was first smoothed using a moving average filter with a window size of 8. The maximum absolute value from after the curve began to level out was then found, and the point at which the energy curve reached 99% or 99.5% of this maximum value was found. This point was considered the approximate beginning of equilibrium. This time was recorded in Table 4 as the beginning time of equilibrium for each energy and each biomarker. The average energy value between this time and the end of the simulation was then calculated using the original, unfiltered data. This average value was also recorded in Table 4 for each energy and each biomarker.

The average value of bond energy indicates the strength of biomechanical bonds and lower the energy higher the likelihood that it will reach equilibrium. An important property of a good biomarker is stability. In this analysis, the time taken to reach equilibrium for each energy is used as an approximate measure of thermodynamic stability. The equilibrium times are compared between the biomarkers for each energy type. Shorter the time a biomarker will be more stable thermodynamically.

Equilibrium electrostatic energy was the fastest for GFAP. However, time for GFAP was insignificant in that it did not fall outside of the 1.5 interquartile range (IQR) limit of the box plot followed by other biomarkers (SF tau and UCH-L1 being the closest Electrostatic energy reached equilibrium the slowest for S100B. Like GFAP, this speed was significant in that the range of equilibrium times was very wide for electrostatic energy, but not outside of the 1.5 IQR range of the box plot. The wide variations in the equilibrium times of all the biomarkers make none of the biomarkers significantly better or worse. The bond energy within 5 ps simulation runs, all the biomarkers presented a similar trend. equilibrium times were very similar across the biomarkers. The equilibrium times was small enough that no biomarker stood alone, that also reflected their applicability. The kinetic, potential, and total energies reached equilibrium for S100B in the shortest amount of time as well.

**Figure 33:**
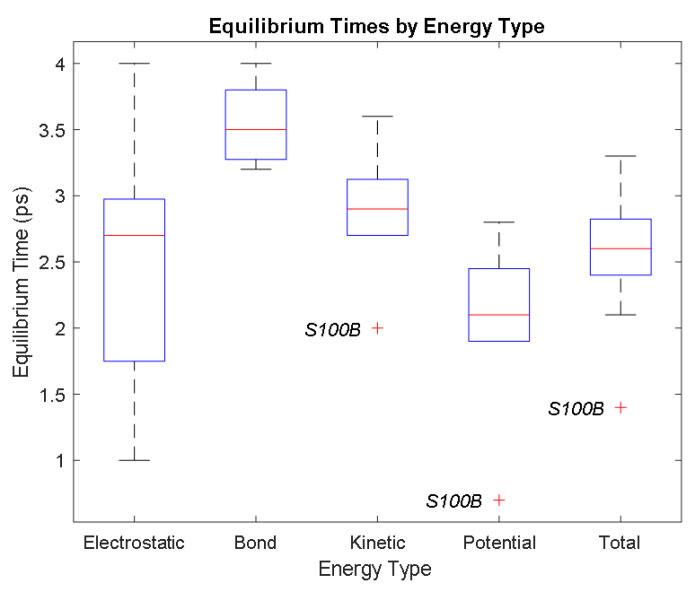
A box plot showing the spread of equilibrium times for each energy type. The outliers were labeled with the names of their corresponding biomarkers.

Overall, S100B was the only biomarker to reach equilibrium significantly quicker than average for any of the energies. This may suggest that S100B may be more thermodynamically stable in aqueous conditions than the other biomarkers. This may support S100B as a potential blood biomarker. However, high thermodynamic stability is a factor that makes it a preferred biomarker. All of the biomarkers simulated were able to reach approximate thermodynamic equilibrium within the timespan of the simulation (5 ps). No biomarker had equilibrium times small enough to suggest that they could not perform well as a blood biomarker. Therefore, thermodynamic stability does not appear to be an issue for any of these biomarkers, and other factors should be investigated to determine the relative utilities of these proteins as blood biomarkers of TBI. The equilibrium time for each energy type was presented in a box plot to show time variations from the medians, Figure 33

**Figure 34:**
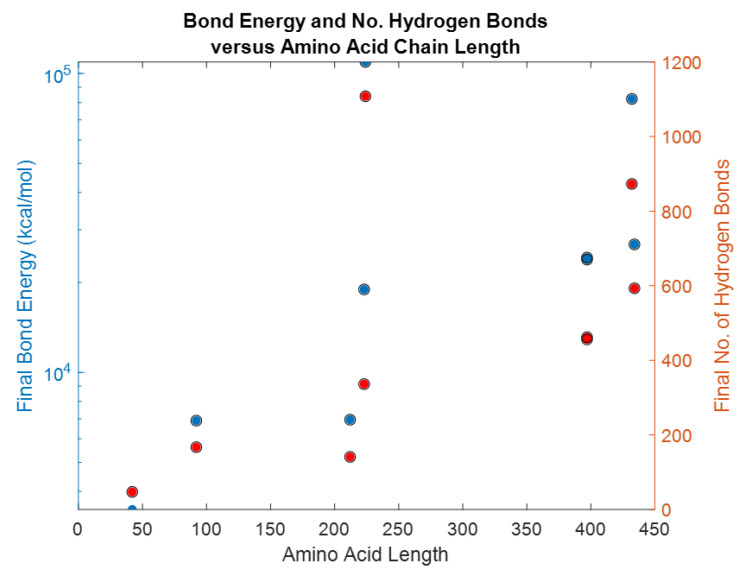
Relationship of number of bonds and bond energy with the amino acid length for the biomarkers.

Each of the simulations for 5ps produced several physical parameters. For a given protein length, the bond energy and number of hydrogen bonds among others, Table 4. It may be possible to hypothesize that biomarkers containing higher bond energy may be able to disrupt the blood-brain-barrier and be detected in the blood flow. There is a significant difference between the bond energy and number of bonds (*p* = 0.0001), however, length of the protein was independent of both bond energy and number of bonds as shown in Figure 34.

## 5. Blood–Brain Barrier

**Figure 35:**
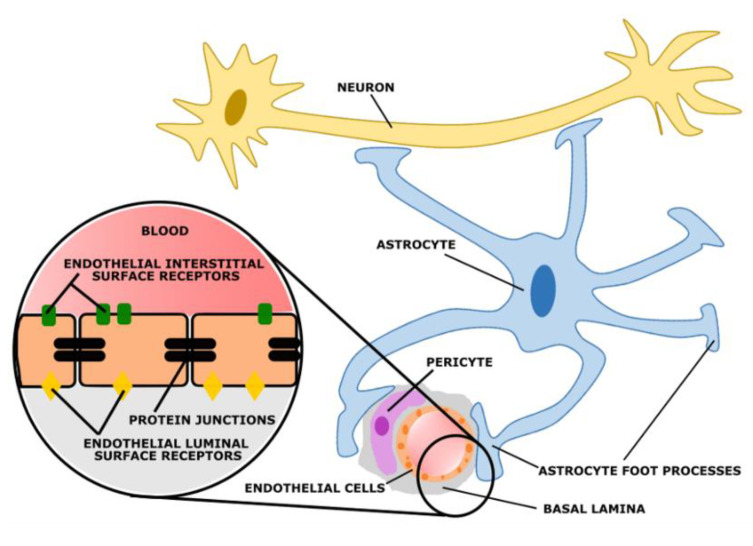
The NVU, highlighting the BBB.

The blood–brain barrier (BBB) is the microvasculature of the central nervous system, designed to not only protect the brain from circulating toxins, but also provide vital nutrients through its selective permeability [90]. Composed of micro-vessels, which are endothelial cells linked by tight junctions, the BBB communicates with neighboring glial cells (astrocytes and microglial) using paracrine signaling [90]. The interactions among the endothelial cells of the BBB and its neighboring cells create a neurovascular unit (NVU), Figure 35. This cerebral hyperaemia, or coupling, is essential for central nervous system homeostasis. Due to the physiological relationships between these cells, they are able to detect neuronal needs and trigger a cascade of signaling (leading to vasodilation or vasoconstriction) to meet its demands [90]. The NVU originates in the basal lamina deep to the endothelial cell monolayer, with tight junction protein complexes spread throughout the endothelial cells to regulate paracellular transport. On the luminal (apical) and basolateral (interstitial) surfaces of the endothelial cells there are cell transporters and receptors to mediate solute and ion transport, which are integral to the NVU. Other cells often included in the NVU are pericytes, smooth muscle cells, neurons, and circulating white blood cells [90].

**Figure 36:**
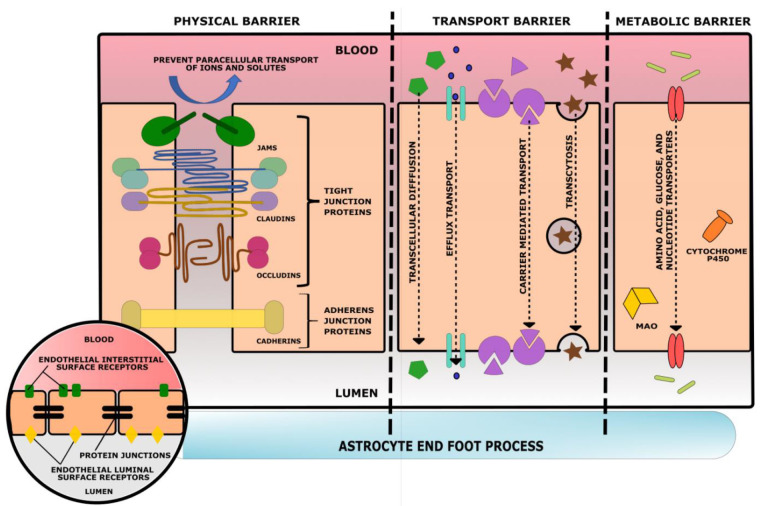
The barriers of the BBB.

The BBB can be divided into three sets of barriers, with each component assigned to a barrier based on its function, Figure 36. The Physical barrier is composed of adherens junction proteins and tight junction proteins to prevent paracellular diffusion of solutes. Adherens junctions are cell-to-cell adhesion complexes that join endothelial cells and aid in proper tight junction formation. Tight junctions, also known as occluding junctions or zonulae occludentes, are multiprotein junctional complexes usually composed of claudins, occludins, and other transmembrane proteins. Zona occludens proteins act as a belt by holding these complexes to the cytoskeleton in order to prevent leakage of solutes between cells. Together, adherens junctions and tight junctions limit any substances’ transport from the bloodstream to the central nervous system as transcellular only. The Transport barrier is composed of ATP-binding proteins, transport proteins, and receptors of endocytosis that promote the transcellular influx of ions and nutrients and the transcellular efflux of toxins. These barrier proteins can be found on the apical and basolateral surfaces of endothelial cells. Lastly, the Metabolic barrier is composed of intracellular and extracellular enzymes (Cytochrome P450, monoamine oxidase, etc.) that metabolize molecules that enter the endothelial cells [91]. These three barriers fortify the BBB and allow for physiological flexibility when the NVU demands it.

Where there is a direct or indirect mechanical force on the brain that results in acute vascular and/or parenchymal change, it is classified as a traumatic brain injury (TBI). In the primary stage of a TBI, contusions, concussions, hemorrhages, hematomas, shearing, or penetrating injuries cause local tissue damage and subsequent breakdown of the BBB. This is followed by a secondary stage of injury, most often inducing cerebral edema, inflammation, hypercapnia, increased intracranial pressure, acidosis, and hyperexcitability, as a direct result of homeostatic imbalance due to BBB injury. Anywhere from hours to days after the primary injury, the compromised integrity of the BBB will initiate a cascade of additional complications that may result in cognitive, motor, perceptual, sensory, communication, language, functional, regulatory, psychiatric, deficits or disturbances [92]. While some brain injuries are mild and symptoms disappear over time, others are more severe and will result in permanent disability if they are not addressed in a timely manner. For this reason, we will focus on tracking specific neural biomarkers (Table 1) in the bloodstream that would be indicative of BBB superpermeability and NVU pathophysiology due to TBIs, allowing for an earlier definitive diagnosis and treatment of TBI.

### 5.1. Effect of Traumatic Brain Injury on the Blood–Brain Barrier

**Figure 37:**
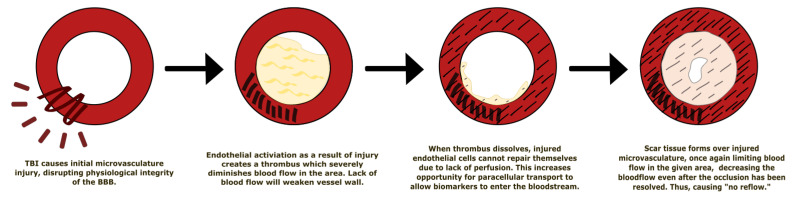
The “no reflow” phenomenon applied to TBIs and the BBB.

Disruption of the structural and physiological integrity of vessels in the BBB leads to endothelial activation of primary homeostasis (platelet plug formation) and the coagulation cascade to stop any hemorrhaging as quickly as possible. This intravascular coagulation leads to microthrombi formation, significantly reduced blood flow, and ischemia—the “no reflow” phenomenon Figure 37 [93]. This phenomenon is defined as “inadequate perfusion through a given segment of circulation without evidence of vessel obstruction” following a temporary occlusion [94]. In other words, when an artery is occluded, detrimental changes (such as swollen intraluminal endothelial protrusion) may occur to the arterioles and capillaries distal to the occlusion [95,96]. When the occlusion is resolved, blood flow to the ischemic tissue may still be impeded, thus having no reflow. With this continued ischemia, the integrity of the BBB is still compromised, allowing factors such as thrombin, albumin, and fibrinogen to enter, which can cause microglial activation, proliferation, and pro-inflammatory factor production.

The BBB is a dynamic structure with fluid permeability that is regulated by the expression of tight junction proteins on the endothelial cells of microvesseles. TBIs cause local tissue damage that disrupts the expression of those proteins and signaling amongst the NVU components. After the initial injury, surrounding astrocytes, pericytes, and microglial respond by releasing molecules that disrupt NVU communication and BBB integrity by decreasing tight junction protein expression. Thus, BBB “openness,” or permeability, is increased, allowing biomarkers to traverse the BBB to get from the brain into systemic circulatory system. Specific pathways taken by the four biomarkers highlighted in this paper (GFAP, NF-L, Tau, UCH-L1) will be discussed individually in the next section.

**Figure 38:**
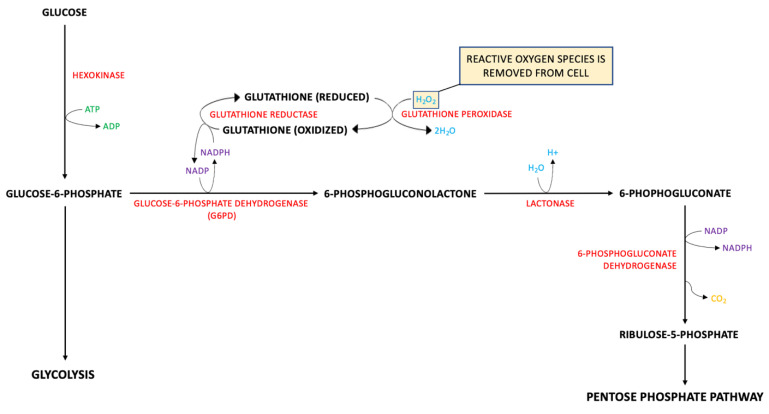
The Pentose Phosphate Pathway is protective for the BBB.

A known contributor to BBB dysfunction after a TBI is oxidative stress. Reactive oxygen species (ROS), such as 4-Hydroxynonenal, are produced by lipid peroxidation of cell membranes after a TBI [97]. As a protective measure against ROS, glutathione is produced as a part of the pentose phosphate pathway (PPP). Glucose-6-phosphate dehydrogenase (G6PD) produces NADPH as a byproduct that is used to reduce glutathione. Glutathione peroxidase combines H_2_O_2_ and the reduced glutathione to create 2H_2_O, thus removing the ROS from the cell Figure 38. Without glutathione, ROS would disrupt tight junction proteins in the BBB, increasing paracellular transport of low molecular weight biomarkers. Glutamine is a precursor for glutathione and glutamate; therefore, a depletion in glutathione is correlated with an increase in glutamate, another molecule that contributes to increased BBB opening. Released from parenchymal neural cells and binding to its mGluR receptor, glutamate increases endothelial permeability [98].

Matrix metalloproteinases (MMPs) are proteins that hydrolyze components of the extracellular matrix and play a role in tissue remodeling, wound healing, angiogenesis, etc. MMP-2 and MMP-9 are gelatinases that degrade basal lamina and tight junction proteins on microvasculature, leading to increased BBB permeability [99]. Tissue inhibitors of MMPs are endogenous protein regulators of MMPs, which decrease MMP expression and induce BBB closing. Deletion of the MMP-9 gene and upregulated TIMPs resulted in decreased brain damage in animal models [100].

Vascular endothelial growth factor (VEGF) is a promoter of angiogenesis that increases permeability in hypoxic conditions by destroying tight junction proteins. The previously discussed ischemia from TBI and the “no reflow” phenomenon provide an ideal environment to induce VEGF expression. VEGFA is a member of the VEGF family and is of note in TBIs, as it is synthesized a few hours after BBB injury. It has been shown to promote BBB opening by downregulating claudin-5 expression and increased vascular endothelial cell permeability via paracellular transport to low molecular weight biomarkers [101,102]. Table 2 shows the molecular weight of biomarkers, only S100B, Ab42, Cortisol and CCL11 are low molecular weight biomarkers, GFAP, UCHL1, NF-L, tau, NSE, CRP and IL-6 are medium molecular weight proteins with the exception of MAP2 which is a high molecular weight biomarker.

When there is vascular wall damage, latent TGF-beta is released from platelets to aid in cell proliferation and differentiation. However, there is limited evidence regarding the true impact of TGF-beta in the BBB post-TBI. Some studies show that TGF-beta is involved in increasing tyrosine phosphorylation, which reduces the expression of tight junction proteins such as claudin-5 and VE-cadherin, increasing BBB permeability. On the other hand, there is evidence that TGF-beta has a role in upregulating N-cadherin, which stabilizes endothelial cell and pericyte interaction in the NVU, maintaining the BBB [103].

Studies show that the degree of permeability of the BBB after a TBI can be evaluated in a number of ways. Dynamic contrast-enhanced magnetic resonance imagining (DCEMRI) has been found to quantify BBB permeability using fast T_1_ mapping to measure the leakage of contrast agent Gadolinium diethylene triamine penta-acetic acid (Gd-DTPA) from plasma into brain. This method is sensitive enough to measure subtle differences in BBB permeability [104].

BBB opening can also be determined by measuring the degree of tight junction protein expression on endothelial cells. Claudin-5 and occludin are such tight junction proteins that are highly expressed on cerebral endothelium and have a key role in regulating paracellular transport in the BBB. Studies on rats show that claudin-5 expression is variable in TBI patients. During an early phase of BBB breakdown, Western blot analyses detected an increase in caveolin-1 expression limited to local tissue damage sites in the NVU. Succeeding the rise of caveolin-1, there was a decreased level of expression of claudin-5 on microvascular endothelium two days after the injury. Occludin expression is biphasic, decreasing on day two and on day four after the injury. Both of these changes in levels of protein expression were limited to the site of tissue damage only [105]. Experiments have shown that claudin-5 expression markedly increases when BBB integrity is restored, about one to two weeks after the injury, and returns to normal levels about three months after the injury [106]. Other confirmed methods of testing BBB breakdown include IgG and Evans blue extraversion. The degree of BBB permeability is dependent on the severity of the TBI, yet any area of local tissue damage will be superpermeable relative to its surrounding, uninjured tissue. This creates an opening for biomarkers to enter the bloodstream, where they can be measured and utilized as indicators of BBB disruption due to a preceding TBI.

### 5.2. Metabolic Pathways of Biomarkers of TBIs

The pathways taken by biomarkers to enter the bloodstream or traverse the blood–brain barrier (BBB) in traumatic brain injuries (TBI) are not fully understood. Though there are many preliminary investigations, there is no definitive accounting for any biomarker’s pathway the brain to the bloodstream or optimal sampling times after a TBI [90,91,92,93,101,102,103,104,105,106,107,108,109,110,111,112,113,114,115,116,117,118,119,120]. We know the BBB is disrupted in moderate and severe TBIs (about 50% of TBIs) [119], but may not be routinely disrupted in mild TBIs, which means there must be more than one route for biomarkers to enter the circulatory system post-TBI. Based on multiple experimental and pharmacological studies, we have proposed the following potential pathways for each biomarker.

#### 5.2.1. GFAP

Glial Fibrillary Acidic Protein (GFAP) is an acidic protein built with 432 amino acids (50 kDa). As a type III intermediate filament, GFAP is the primary contributor to cell cytoskeletons to provide mechanical support in the plasma membrane of the BBB [107]. Since it is an astrocyte-specific marker in the central nervous system (CNS), GFAP maintains astrocyte stability and helps create the BBB. Astrocytes, as discussed in previous sections, are a crucial part of BBB structure formation and maintenance. While endothelial cells form the BBB and tight junction proteins hold individual endothelial cells together to prevent paracellular transport, astrocytic end-foot processes maintain the structure of endothelial cells as a whole. Additionally, astrocytes secrete molecules that promote cell-to-cell communication and strong tight junction formation in the BBB [108]. This makes astrocyte stability, and by extension GFAP availability, paramount in BBB functioning.

**Figure 39:**
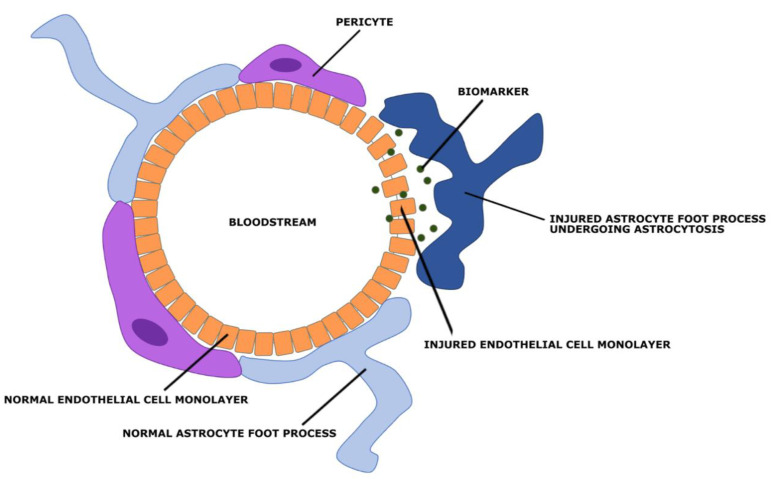
Astrocytic damage degrades the integrity of the endothelial cell monolayer, allowing for increased paracellular transport of biomarkers through the BBB.

Under normal conditions. GFAP expression is controlled by the Jak-STAT signaling pathway and has relatively stable levels in blood. Yet, during CNS pathology, there is damage to neurons and glial cells, such as astrocytes. As a response, GFAP levels are elevated due to increased expression of GFAP mRNA in efforts to recover astrocyte stability [109,110]. Local tissue damage in TBI does not allow astrocytes to immediately recover, causing reactive gliosis, in which GFAP spills out of injured astrocytes. Moreover, astrogliosis weakens interactions between astrocyte end-foot processes and endothelial cells of the BBB (Figure 39). It has been shown that astrocytes are heavily involved in regulating vasodilation and vasoconstriction of these cells [108]. Without proper astrocyte functioning, there are more openings between endothelial cells and weaker tight junction proteins. Thus, the combination of astrocytic damage and increased intracellular levels of GFAP allow the opportunity for paracellular transport of GFAP across the BBB. This leads to an elevated level of GFAP in the blood, which can be measured and compared to baseline levels to use as a diagnostic for TBI and BBB injury.

#### 5.2.2. NF-L: There Are Four Major Proteins Involved in the Formation of Neurofilaments

Alpha-internexin (a-int), heavy (NF-H), medium (NF-M), and light (NF-L). All are type IV intermediate filaments, but neurofilament light chain protein is the most abundant of the four. NF-L is built as subunits of cylindrical proteins with 310 amino acids (60 kDa), exclusively in neuronal cytoplasm. Expressed highly in large-calibre myelinated axons, as well as in neuronal cell bodies and dendrites, NF-L has an important role in the structure and support of neurons [111].

Under normal conditions, there is a consistent release of NF-L from axons which naturally increases with age. However, under pathologic conditions, there is CNS damage that extends to the axons of neurons, where there will be consequential demyelination. Without a protective myelin sheath, NF-L is exposed to interstitial fluids and cerebral spinal fluid (CSF). As levels of NF-L drastically increase, it is phagocytosed and degraded by CSF cells [112]. The debris is drained into local lymph nodes at the cribriform plate, where the lymph fluid is returned to the bloodstream via the subclavian vein. This biomarker may not require injury to the BBB for there to be an increase in the level of NF-L found in CSF and blood. NF-L is a highly sensitive, yet unspecific, marker of axonal injury. This is not a concern for our purposes because any TBI would cause neuronal axon damage and release of NF-L into the circulatory system. In fact, not only does NF-L have the potential to be a diagnostic marker for TBI, the level of NF-L in blood samples can also be used to predict severity of TBI.

#### 5.2.3. Total Tau

Tau is a basic, hydrophilic microtubule binding protein with six major isoforms (ranging molecular weights). Total tau (t-tau) is the measure of all phosphorylated (p-tau), non-phosphorylated, cleaved (c-tau), and non-cleaved tau isoforms. As a microtubule-associated protein, tau regulates microtubule activity and cytoskeleton stability, specifically in elongated neuronal axons of the central nervous system [107]. Highly expressed in thin, non-myelinated axons of interneurons, tau is a neuron-specific marker of CNS injury [113]. Ratios of isoforms of tau have been investigated as markers for TBI, but our review focuses on t-tau alone.

Physiologically, tau is released by healthy neurons into interstitial fluid and moves across the BBB. Studies show that caveolin-1, a protein expressed in neural endothelial cells, is integral in tau transport through the BBB [114]. However, in response to CNS damage, caveolin-1 expression is decreased, forcing tau to accumulate in cerebral interstitial fluid. From there, tau is phosphorylated and degraded into smaller fragments, to be phagocytosed by CSF cells. Just is NF-L, those small bits of protein debris are drained by the lymphatic system and return to the bloodstream. The elevation in these tau monomers can be measured in blood serum as a predictor of neuronal injury and TBI.

#### 5.2.4. UCH-L1

Ubiquitin Carboxy-terminal Hydrolase-L1(UCH-L1) is a thiol protease built with 223 amino acids (24 kDa). Primarily found in neurons, UCH-L1 plays a role in neuronal repair after injury by targeting excessive, oxidized, or misfolded proteins for catabolism through the ATP dependent ubiquitin-proteasome pathway [115]. It can ligate ubiquitin onto proteins as well as hydrolyze ubiquitin from proteins, generating free monomeric ubiquitin and protecting neurons from injury.

As a neuron-specific marker that maintains axonal integrity, UCH-L1 is similar to NF-L. Released at low levels under physiologic conditions, UCH-L1 levels are significantly increased in CSF and blood after a TBI. Once there has been damage to the central nervous system, axonal injury promotes UCH-L1 activity and proliferation. In turn, there is an excess of UCH-L1in the interstitial fluid, which removes such waste products from the CNS into the CSF. Then, UCH-L1 is phagocytosed by CSF cells, drained into lymphatic fluid, and returned to the circulatory system, where it would be renally excreted. Nonetheless, there is one major difference between NF-L and UCH-L1: size. UCH-L1 (24 kDa) is much smaller than NF-L (60 kDa). This may allow for a secondary method for UCH-L1 to enter the BBB. With the buildup of UCH-L1 in the extracellular space and its smaller size, UCH-L1 may be able to pass through the BBB paracellularly as neuronal injury weakens tight junction proteins between endothelial cells (Figure 38) [116]. Studies show a stark increase in UCH-L1 levels in CSF, so we suspect lymphatic drainage is the primary pathway of UCH-L1 to the bloodstream but directly crossing the BBB may be a minor pathway involved in the process. After it has been released into the circulatory system, UCH-L1 levels can be measured from blood samples and compared to baseline measurements to predict TBI.

## 6. Kinetics of Biomarkers in TBI/CTE/PTSD

Time-concentration plots need to be constructed at different intervals to assess the biomarker concentration in fluids such as blood, serum, plasma and cerebrospinal. Several trends appear from kinetic study including time to peak concentration and half-life. One-compartmental pharmacokinetic model had been used with two first order rate constants absorption and elimination, to predict the biomarker level at a given time [117]. Kinetic modeling allows determination of biomarker concentration in blood if the other parameters such as rate of biomarker released and absorbed, and volume of distribution known for a given biomarker. As a result, biomarker concentrations are complex kinetic profiles, which do not follow normal kinetic parameters but may have certain peak and decay rates with mild predictability [118]. Another confounding factor is total blood volume, more blood volume will cause a decrease in biomarker concentration [119]. Kinetic parameters include peak or t_max_, the time to maximum concentration, while half-life, or t_1/2,_ is time for biomarker concentration to decrease to 50% of maximum concentration [119]. Kidney filtration affected by age or disease may alter the kinetics of elimination as well. Metabolic kinetics may include other models, [120] the law of mass action, describing the quantitative aspects of a chemical reaction under ideal conditions. If a substance *C* is formed by the reaction of substance *A* and substance *B*, the production of *C* can be described by the following equation product *C* = *k* ∗ *A* ∗ *B* where *A*, *B*, and *C* are concentrations changing over time, and *k* is a rate constant describing the speed of the reaction.

Michaelis-Menten rate law (MMRL) introduced by Michaelis & Menten [121]

$$v=\frac{Vmax*S}{Km+S}$$

where *v* is the reaction rate, *V_max_* the maximum reaction rate, *S* the concentration of the substrate, and *K_m_* the Michaelis constant (the substrate concentration at half of the maximum reaction rate).

The Michaelis-Menten model describes the reaction kinetics of a single-substrate reaction, in which the conversion of a substrate *S* into a product *P* takes place via the formation of an intermediate complex *ES*, where *k_1_*, *k_2_* and *k_3_* denote reaction rates.

Biochemical systems tend to remain in homeostasis, which is described by the equilibrium constant [121]

$$K_{eq}={\left[C\right]}^{c}{\left[D\right]}^{d}/{\left[A\right]}^{a}{\left[B\right]}^{b}$$

*K_eq_* the equilibrium constant in the general reaction *aA + bB*↔*cC + dD*, where *a*, *b*, *c*, *d* are the number of molecules of *A*, *B*, *C*, *D* participating, and [*A*], [*B*], [*C*], [*D*] are the molar reaction concentrations of the reaction components at equilibrium.

Due mainly to limited number of studies were available in the literature describing the concentration differences with time after injury it was not possible to develop kinetic models. Certain biomarkers were extensively studied, such as S100B and GFAP, whereas others have been reported sparingly. Additionally, the mechanism for biomarker release into the blood is not completely understood through a standard pathway. Proposed methods include disruption of the blood–brain barrier, axonal injury, neuroinflammation, or routing through the glymphatic system [118,122,123,124,125]. Furthermore, factors like clearance, elimination, time since injury, patient age related variability affect biomarker kinetics.

### 6.1. Kinetic Parameters of Selected Biomarkers

Data compiled from the literature included the following parameters, tabulated below.

### 6.2. Biomarker Kinetics

Although the biochemical markers have been successfully used to diagnose other diseases, their use in the TBI has been not as successful due to TBI conditions, varying from mild to severe and transitioning to PTSD and/or CTE. Biomarkers that are elevated during the acute phase of the severe trauma are tabulated below. This study only uses data post injury in the rise of markers.

Kinetics of biomarkers discussed further include S100B, GFAP, UCHL1, NF-L and tau. Each of these protein structures are discussed in the homology, molecular dynamics simulations of protein structures forming bonds with hydrogen, their bond energy, and other physical and structural parameters (Table 2 and Table 4, respectively).

#### 6.2.1. S100B

**Figure 40:**
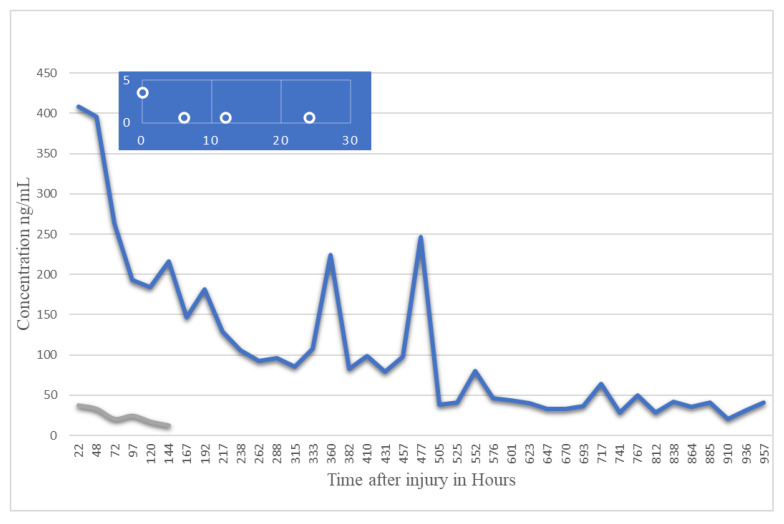
Kinetics of biomarker S100B after the injury data from [119,126,138,139,140].

S100B is generally found to gradually decrease after trauma like other biomarkers. Several studies have indicated a correlation between initial S100B levels and TBI severity. There has been a lot of variability in S100B studies, with some indicating a rapid decline in the protein’s serum levels while other studies note a more gradual decline. Multiple studies have reported a second peak post-trauma, approximately 48 h after the initial trauma (noted in Table 5 and Table 6). The half-life of S100B appears to vary based on the severity of TBI, with a mild TBI displaying a S100B half-life of 2–6 h while a severe TBI results in an S100B half-life of approximately 24 h, Figure 40. Samples would best be recovered between 1 and 3 h after TBI [119]. Controversial and limited due to low specificity [126].

#### 6.2.2. GFAP

**Figure 41:**
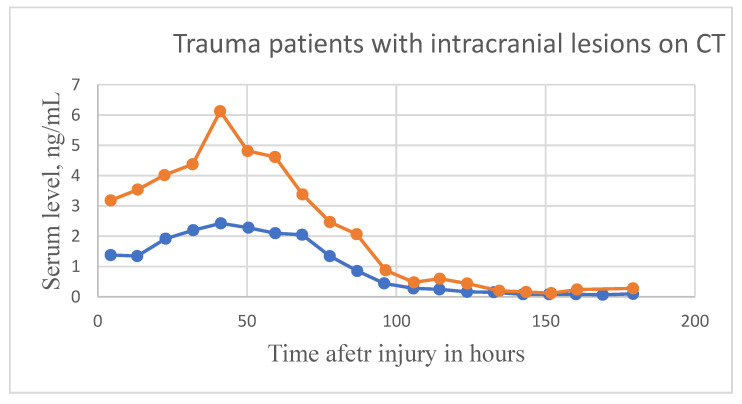
Temporal profiles of GFAP in trauma patients with mild or moderate TBI with surgical intervention and blue line indicates the presence of lesion in CT (data from [141]).

GFAP concentration levels remain elevated for approximately 7 days, but generally have a trend to decline overtime, Figure 41. Peak concentration is noted to be around 20 h post-injury, with an estimated half-life of 24–48 h. Best sampling time would be between 6 and 18 h [119]. Conflicting studies [127] indicate GFAP a good biomarker for TBI detection, specifically due to the lengthy half-life. Good specificity and sensitivity, however other studies reported increased GFAP after orthopedic trauma, indicating a possible lack of selectivity [131,132,136].

#### 6.2.3. UCH-L1

**Figure 42:**
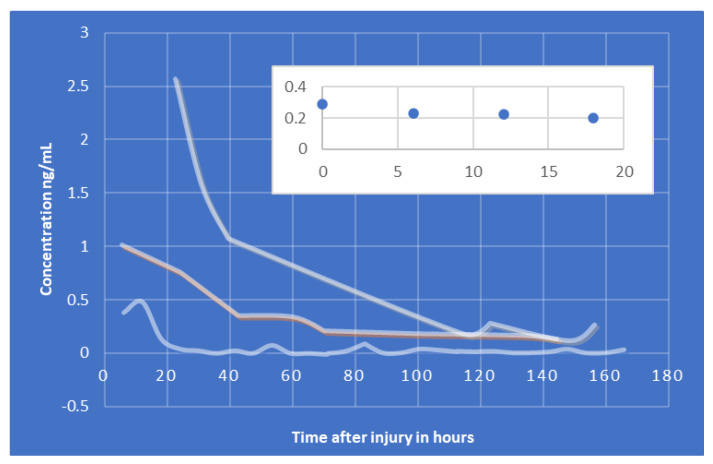
Kinetics of biomarker UCH-L1 (data from [141,142,143]).

UCH-L1, a protease involved in the removal of ubiquitin in neurons, appears to peak around 8 h post-injury, and a half-life of around 6 h for mTBIs (slightly longer for more severe cases) [141]. The serum levels UCH-L1 generally appear to decline overtime. Optimal sampling time post TBI would be 2–8 h [119]. Good indicator, Figure 42, for poor outcomes [136]. Noted it was best used for early trauma cases, with decreasing accuracy with time [134,141,142].

#### 6.2.4. NF-L

**Figure 43:**
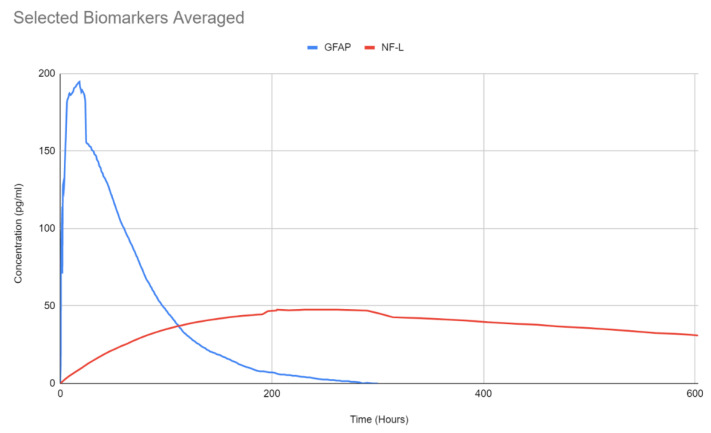
Average concentration data plotted for GFAP and NF-L showing NF-L stays elevated for longer duration (data from [119]).

**Figure 44:**
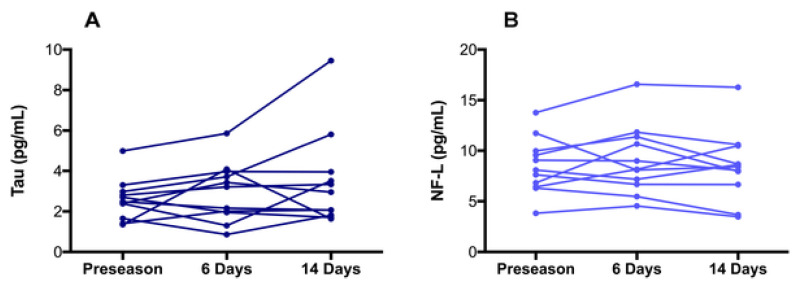
Kinetics of Tau (**A**) and NF-L (**B**) (data from [144]).

In the case of neuronal/axonal injury NF-L has been investigated in the literature. Contrary to previous biomarkers, NF-L levels appear to rise over time, Figure 43 and Figure 44B. There are very few studies that focused specifically on NF-L, however, they appear to indicate an increase of the protein for up to 2 weeks. Elevated levels of NF-L have been reported up to a year post TBI [119]. NF-L concentration can vary with physiologic changes such as pregnancy, BMI, cardiovascular health, diabetes, or other diseases. Consequently, it may have limitations differentiating TBI from other conditions [135] as a result sports related concussion data was used from literature to show the trend (see Figure 44B). Similar data are reported as a part of CARE program, however, not used in this paper. The GFAP and NF-L composite plot is shown below.

#### 6.2.5. Total Tau

**Figure 45:**
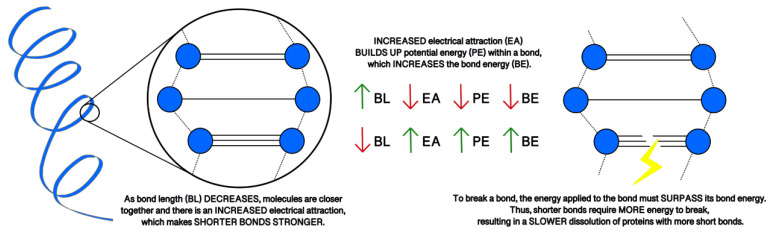
Structure of biomarkers and kinetics relationship.

**Figure 46:**
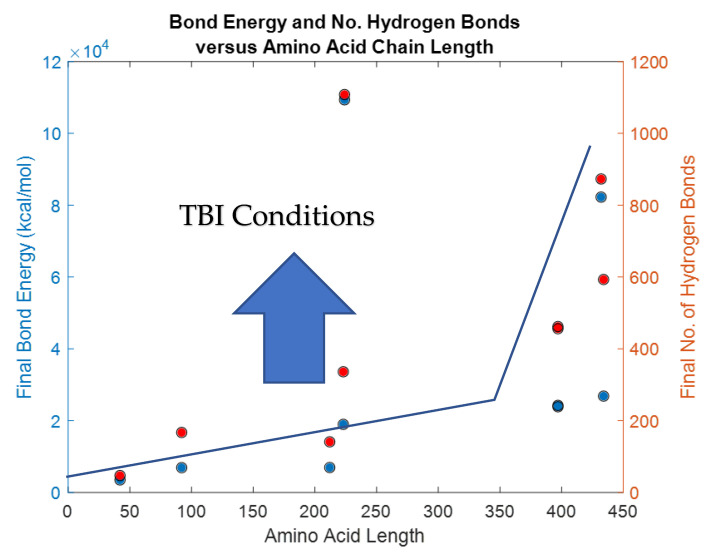
For a given time of dynamic simulation, bond energy and number of hydrogen bonds formed by the proteins with different amino acid length may indicate biomarker viability in diagnosis, blue line showing lower bound region where biochemical reactions stop.

**Figure 47:**
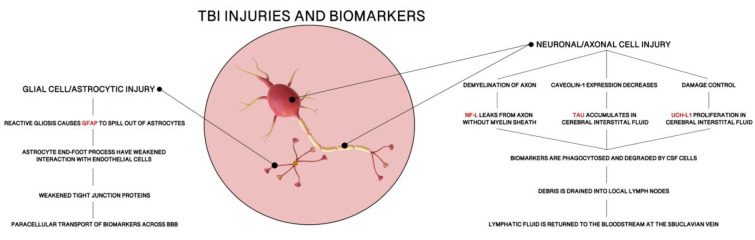
Pathway for biomarkers (GFAP, UCHL1, tau and NF-L) studied in this research all involving injury at cellular and neuronal/axonal levelS100B is presented below. Since this work is under development for a future article in Bioengineering, we will not discuss the format in which the data presentation will be carried out. Figure 40 shows a typical kinetic plot for S100B, data shows rapid decline in the concentration after 2 weeks while the half-life is before one-week after injury. Normalized data presented in terms of concentration with respect to time in Figure 48A. Attempt was made to reorganize the data in a new format in Figure 48B showing the concentration gradient with respect to time after injury. This behavior will be modeled by Michaelis-Menten equation [121].

Tau is a CNS-enriched microtubule associated protein expressed in thin, unmyelinated axons forming their stability. These tightly formed bonds lead to a slower initial dissolution of the protein, as explained in Figure 45 and Figure 46 and Table 7. Elevated levels of tau have been observed up to a year after TBI [119] and found to increase in CSF following injury resulting in acute TBI. A summary of this protein’s proposed pathway can be seen in Figure 47. Plasma levels of total tau remain elevated for 12 days following concussion in professional sports [143,144,145] with the highest concentration measured after 1 h post injury. The concentration diminishes within 24–72 h post injury and may be lower than non-concussed athletes [144]. As stated for NF-L, other conditions such as exercise may elevate the total tau and need to be accounted for in the protocol. The two concentrations, NF-L and Total tau, presented below, PLOS-ONE.

**Figure 48:**
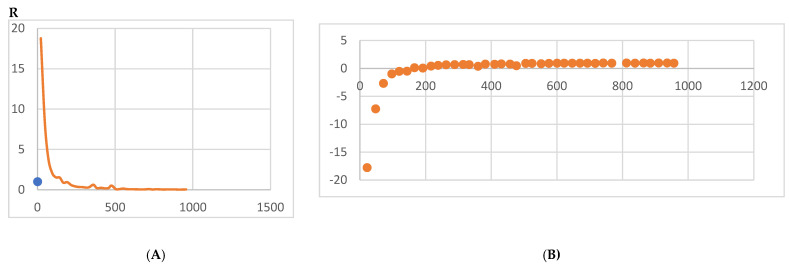
Left, (**A**) showing normalized concentration with respect to time for S100B, Figure 40 data, (**B**) showing a plateau in the concentration after 200 h of injury which is detectable indefinitely, (horizontal axis of 1000 h).

The proposed pathways for each of the discussed biomarkers are summarized in Figure 47 [146,147,148,149,150,151]. It is evident from the Figure 48 that such presentation of TBI kinetics data will prove valuable in clinics in the diagnosis and render needed treatment or rehabilitation. Since 800 h of data shows nearly a linear, constant behavior, within this range TBI is detectable, not possible before.

In 2018, the FDA approved the use of blood tests to detect brain tissue damage, especially for injuries that are not measurable by CT scan, to avoid unnecessary radiation exposure for patients. The primary proteins utilized in this blood test are UCH-L1 and GFAP, which are measured within 12 h of head injury [152]. This window of time is significant, as other studies that broadened the window to 24 h yielded results with extremely low sensitivity [153]. These biomarkers show an acute rise in serum concentration due to neuronal cell body injury and gliosis within minutes of TBI, followed by a sharp decline in levels within hours of injury (particularly UCH-L1). One study showed that GFAP levels reached a peak at 20 h and UCH-L1 reached a peak at 8 h after injury [154,155]. The level of serum protein reliably predicts the presence or absence of intracranial lesions visible by CT scan; it does not detect the severity of TBI. Some studies show that GFAP outperforms all other biomarkers and can detect MRI abnormalities in CT-negative patients. Yet, using both biomarkers in conjunction provides unique benefits as GFAP levels are high in patients with mass lesions (polytrauma), whereas UCH-L1 levels are higher in patients with diffuse injury [155].

The Banyan Brain Trauma Indicator (BTI) was evaluated by the FDA based on a clinical study of 1947 individuals (across 22 clinical sites in 3 countries) with suspected mTBIs, whose blood test results were compared with CT scan results. If the concentration of proteins is above the predetermined upper limit of quantitation, the result is reported as “Above” the cutoff value. If the concentration is below the lower limit of the lower limit of quantitation, the result is reported as “Below” the cutoff value, establishing semi-quantitative results. For UCH-L1, “Above” is a concentration of 327 pg/mL or above and “Below” is any concentration less than 327 pg/mL. For GFAP, “Above” is a concentration of 22 pg/mL or above and “Below” is any concentration less than 22 pg/mL [152,156].

However, there are still many application limitations for utilizing the BTI. The blood draws must be taken within the first 12 h of injury. When the data is broken down into 4-h intervals, it can be seen that the sample size for the 8–12 h group is significantly smaller (*n* = 84) than the other groups, making the data increasingly unreliable for that time frame [156]. Thus, samples must be taken as early as possible to maximize accuracy. Additionally, patients with pre-existing neurodegenerative diseases (Alzheimer’s disease, Parkinson’s disease, Creutzfeldt-Jakob disease, ALS, Guillain-Barre syndrome) will already have an elevated serum baseline of TBI biomarkers, yielding in a higher rate of false positives with the BTI. The issue of false positives is further supported by the clinical performance results of the BTI. Boasting a sensitivity rate of 97.5%, the BTI only has a 36.5% specificity rate. Moreover, the test has a negative predictive value of 99.6%, but a positive predictive value of 9.2% [156]. With such a high rate of false positives, is this procedure clinically significant in preventing exposure to unnecessary radiation? While the benefits may outweigh the costs, the Banyan BTI also includes the dangerous risk of false negatives (2.5%); thus, it must be used as an adjunct to diagnostic testing rather than a stand-alone device [156]. Banyan’s BTI requires skilled technical personal to operate, takes several hours to run, and has not been commercialized, so this device is not widely available in the clinical setting. There are other devices in development (iSTAT) that, pending FDA clearance, will be widely accessible in clinical setting across America [155].

## 7. Conclusions

The equilibrium times are compared between the biomarkers for each energy type. Shorter the time a biomarker will be more stable thermodynamically. Equilibrium electrostatic energy was the fastest for GFAP. However, time for GFAP was insignificant in that it did not fall outside of the 1.5 IQR limit followed by other biomarkers SF tau and UCH-L1 being the closest. Like GFAP, this speed was significant in that the range of equilibrium times was very wide for electrostatic energy, but not outside of the 1.5IQR range. The wide variations in the equilibrium times of all the biomarkers make none of the biomarkers significantly better or worse. The bond energy within 5 ps simulation runs, all the biomarkers presented a similar trend. Equilibrium times were very similar across the biomarkers. The equilibrium times was small enough that no biomarker stood alone, that also reflected their applicability. The kinetic, potential, and total energies reached equilibrium for S100B in the shortest.

The kinetic, potential, and total energies reached equilibrium for S100B. Equilibrium times were also considered outliers for each of these energies, in that S100B was significantly faster than all the other biomarkers (greater than 1.5IQR away from the mean). The slowest times for all three of these energy types were not considered significantly different from average.

Overall, S100B was the only biomarker to reach equilibrium sooner than average for any of the energies. This may suggest that S100B may be more thermodynamically stable in aqueous conditions than the other biomarkers. This may support S100B as a potential blood biomarker. However, high thermodynamic stability is a factor that makes it a preferred biomarker. All of the biomarkers simulated were able to reach approximate thermodynamic equilibrium within the timespan of the simulation (5 ps). No biomarker had equilibrium times small enough to suggest that they could not perform well as a blood biomarker. Therefore, thermodynamic stability does not appear to be an issue for any of these biomarkers, and other factors should be investigated to determine the relative utilities of these proteins as blood biomarkers of TBI.

BBB permeability is a function of Vascular endothelial growth factor (VEGF). VEGFA is a member of the VEGF family and is of note in TBIs, as it is synthesized a few hours after BBB injury. It has been shown to promote BBB opening by downregulating claudin-5 expression and increased vascular endothelial cell permeability via paracellular transport to low molecular weight biomarkers. The molecular weight of biomarkers, S100B, Ab42, Cortisol and CCL11 qualify for low, GFAP, UCHL1, NF-L, tau, NSE, CRP and IL-6 are medium with the exception of MAP2 which is a high molecular weight biomarker. However, clinically only one low molecular weight biomarker, S100B, was found in the blood and detected for potential TBI condition.

The concentration of biomarkers, post injury, rapidly rises and then reaches a plateau. The half-life of some of these biomarkers range from 6 h to 24 h. Slight variations are possible as the mitigation strategies are developing that allow blood testing immediately after the injury along with required CT/MR imaging. Abnormal head scan along with elevated biomarker are together indicated for mild to moderate TBI in sports, fall and military injury. Even though most biomarkers concentration deplete with time, NF-L has been shown to rise for about two weeks after injury. While tau also shows such a trend, though it interacts with activities of daily living and therefore not a reliable biomarker. A new framework of presenting kinetics data shows great promise in clinical settings.

Molecular dynamics models presented with bond energy and number of hydrogen bonds formed during the period of 5ps simulations. For a given protein length, the bond energy and number of h-bonds show a lower bound within which the biomarker kinetics were not measurable. However, above the lower bound parameters some success was found in this research where GFAP was an outlier, meeting our hypothesis. The empirical data presented may help future discovery of TBI/PTSD/CTE biomarkers.

In conclusion, all of these biomarkers presented in this paper are indicative of TBI. However, it is clear that the mechanism of TBI from external forces, forward wave force and shrapnel generate tissue damage rupturing the BBB expressing the biomarker in the blood stream. Some of the clinical studies used S100B, GFAP, UCHL1, NF-L and tau with limited diagnostic success. Each biomarker has a unique role, domain, and pathway to reach the bloodstream. If the TBI is neuronal in nature, NF-L, Tau, and UCH-L1 will be more accurate predictors because they enter the bloodstream through cerebral spinal fluid and lymph nodes. Yet, if the TBI is astrocytic in nature, GFAP and S100-B will be the better biomarkers because they enter the bloodstream by directly crossing the BBB.

## Figures and Tables

**Table 1:** Summary of the biomarkers [5,6,7,8,9,10,11,12,13,14,15,16,17] present in TBI conditions, PTSD and CTE.

Biomarker	Condition	Findings
GFAP	TBI	Excellent indicator of TBI as well as abnormal CT or MRI findings [7,8].
UCH-L1	TBI	Very good early predictor of TBI and abnormal CT findings, [8].
MAP-2	TBI	Early levels have limited predictive ability of abnormal CT results but good predictive ability of early mortality[5]
NF-L	TBI	Good indicator of TBI and abnormal CT findings [6].
Total tau	TBI	Decent indicator of abnormal CT findings [9].
S100B	TBI	Not specific to TBI and not indicative of abnormal CT [8].
Aβ42	TBI	Decent indicator of mTBI but not correlated with post-concussive or behavioral symptoms [10].
NSE	TBI	Good indicator of TBI but not directly correlated with TBI severity [11,12].
CRP	TBI	Non-specific to TBI, but good predictor of 6-month neurological outcome after TBI [13,14]
IL-6	TBI and PTSD	Correlated with number of previous TBIs and with PTSD symptoms in people with repetitive TBIs[15]
Cortisol	PTSD	Levels, not correlated with either TBI/PTSD/CTE, significantly correlated with a history of trauma[16]
CCL11	CTE	Limited evidence of strong differentiation between CTE subjects and non-CTE subjects, but not associated with CTE severity [17].

**Table 2:** The molecular weight, amino acid length, and basal isoelectric point (the pH value at which it is electrically neutral) of the identified biomarkers.

Biomarker	Weight (kDa)	Amino Acid Length	Basal Isoelectric Point
GFAP [24,46]	49.88	432	5.42
UCH-L1 [26,47]	24.824	223	5.33
MAP-2 [28,48]	199.526	1827	4.82
NF-L [30,49]	61.517	310	4.64
Total tau [50,51]	36.7–49.5	352–441	~6.3
S100B [34,52]	10.713	92	4.5
Ab42 [36,53]	4.514	42	5.5
NSE [38,54]	47.269	434	4.91
CRP [40,55]	25.039	224	5.45
IL-6 [42,56]	23.718	212	6.17
Cortisol [43]	0.362	N/A	--
CCL11 [45,57]	10.732	97	9.98

**Table 3:** Overall Biomarkers found from previous clinical trials [71,72,73,74,75,76,77,78,79].

Biomarkers	Condition	Findings
miRNA (plasma)	TBI	There was a significant elevation of miRNAs in the TBI study population. Three miRNAs may be particularly useful in identifying chronic TBIs [71].
miRNA (EV)	TBI	Potential indicator of chronic blast-related TBI, dozens of miRNA identified but needs further investigation [71].
CRP (plasma)	TBI	Indicator for TBI, shown through iTRAQ and validated with ELISA [71].
MME (plasma)	TBI	Potential indicator for chronic mTBI, strong indicator in long-term TBI patients.
Cortical thinning (imaging)	TBI	Strong indicator to differentiate between blast-related TBI and non-blast-related TBI. However, this is still a neuroimaging biomarker [72].
NRGN (blood)	TBI	Potential indicator of mTBI in pediatric patients [73].
S100B (blood)	TBI	Limited diagnostic use for mTBI patients due lack of specificity; increased levels in multiple extracranial pathologies [73,74]. Serum concentration in blood samples drawn less than 3 h after trauma is an accurate predictor of a normal CT scan for mTBI patients [79]
GFAP (blood)	TBI	Not specific enough as a diagnostic indicator due to high levels in multiple types of brain injuries [73] Greater indicator than S100B, especially for injuries with delayed treatment such as military personnel in combat situations [72] Additional trial in progress [79]
Metabolic Panel ((FA 2-OH C16:0, FA C18:0, TUDCA, PE ae C36:4, PE aa C38:6, and LysoPC a C20:4)	TBI	Potentially strong indicator of mTBI for recent injuries up to 7 days post injury [75]
FDG on PET (imaging)	TBI	Indicator of neuronal activity; number of blast exposure correlates with FDG uptake in veterans [76]
P-tau on PET(imaging)	CTE	Neuropathological evidence *correlating* p-tau in an irregular pattern to ante-mortem cognitive and neuropsychiatric symptoms of CTE [77]
P3b ERP (neurologic testing)	TBI + PTSD	Diminished P3b amplitude during DS-CPT is a strong potential indicator of blast-related mTBI and/or PTSD after combat trauma, but is unable to differentiate between mTBI and PTSD [78]

**Table 4:** The times at which each energy (electrostatic, bond, kinetic, potential, and total energies) reached equilibrium, and the average energy values during equilibrium for each biomarker.

		Electrostatic	Bond	Kinetic	Potential	Total
Aβ42	Time (ps)	2.7	4	2.9	1.9	2.8
	Avg. Value (kcal/mol)	−32,784.33	3480.92	7455.66	−23,434.85	−15,997.53
CRP	Time (ps)	2.2	3.2	3	2.4	2.6
	Avg. Value (kcal/mol)	−1,019,308.27	109,340.86	232,063.93	−723,307.82	−491,584.45
GFAP	Time (ps)	1	3.3	3.1	2.8	2.9
	Avg. Value (kcal/mol)	−823,609.83	82,245.14	173,754.06	−605,439.31	−431,757.93
IL-6	Time (ps)	2.9	3.8	3.6	2.1	3.3
	Avg. Value (kcal/mol)	−64,264.60	6949.12	14,990.33	−45,343.51	−30,407.70
NSE	Time (ps)	2.9	3.7	3.2	1.9	2.5
	Avg. Value (kcal/mol)	−241,850.97	26,823.98	57,205.73	−168,420.32	−111,428.98
PHF Tau	Time (ps)	3.2	3.8	2.7	1.9	2.1
	Avg. Value (kcal/mol)	−213,881.08	24,239.10	51,936.30	−146,812.80	−95,005.53
S100B	Time (ps)	4	3.5	2	0.7	1.4
	Avg. Value (kcal/mol)	−65,958.61	6900.85	14,928.18	−46,223.02	−31,437.77
SF Tau	Time (ps)	1.6	3.4	2.7	2.6	2.6
	Avg. Value (kcal/mol)	−209,627.89	23,856.03	51,337.40	−144,155.15	−92,829.63
UCH-L1	Time (ps)	1.8	3.2	2.9	2.4	2.7
	Avg. Value (kcal/mol)	−175,430.48	18,957.84	40,291.56	−123,831.34	−83,573.74

**Table 5:** Kinetic parameters of select blood/serum/plasma-based biomarkers.

Biomarker.	Detectable	Peak	Longevity	Half-Life	Order	References
S100B		1st: ~6 h 2nd: ~48 h	Gradual decline over first 48 h, 2nd peak then subsequent decline ~96 h	~2–6 h mTbI ~24 h severe TBI	First-order, exponential decay *	[122]
GFAP	<1 h	~20 h	~168 h (7 days)	24–48 h		[122]
UCH-L1	<1 h	~6–8 h	Gradual decrease over 48 h	7–10 h		[122,125,126,127]
NF-L	~6 h **		Continuous increase for up to 10 days ***			[128]
Total Tau	<1 h	<1 h	Steady decline over 12 h, levels detectable up to 18 months	~36 h		[122]
MAP-2	~6 h	N/A	Stable for 24 h			[129,130]
CCL-II		~24 h				

* Conflicting information in various studies, complex kinetics; ** Study found NF levels detectable within 6 h in animal trial, not currently known for humans [128]; *** Only few studies have investigated NF-L kinetics.

**Table 6:** Biomarker origin, fluid source, indications, and comments specific to TBI.

Biomarker	Origin	Extra Cerebral Source	Sample Source	Indication	Comments	References
S100B	Astrocytes	Adipocytes, chondrocytes, cardiac and skeletal muscle, melanoma	CSF, blood serum	mTBI, BBB disruption	Not specific enough to TBI	[118,122,126]
GFAP	Astrocytes Fibroblasts	N/A	CSF, serum	Increased with intracranial pressure,		[122,131,132]
UCH-L1	Neurons	Lung tumors, testis/ovaries	CSF	Breakdown of BBB, serum levels correlated to severity of injury and mortality	Potential for prognostic and diagnostic use	[122,126,129,133]
NF-L	Neurons (axon)	N/A	CSF, blood serum	Neural death, axon disintegration, severe TBI	Potential long-term indicator; age, diabetes, BMI, and pregnancy noted to alter levels of NF-L	[126,134,135]
Total Tau	Neurons (axon), Astrocytes	N/A	CSF, blood serum	Severity of injury and mortality	CSF appears to be more accurate than blood serum	[122,126,134,136]
MAP-2	Dendrites			Dendritic injury	Severe TBI patients had high levels present after 6 months	[130,137]
CCL-II						

**Table 7:** Dynamic simulations of TBI biomarkers showing specific trends for possible use in TBI prediction [140,141,142,143,144,145,146,147,148,149,150,151].

Biomarker	Homology Figures	MD Simulations Figures	Metabolic pathway BBB	Bond Energy kcal/mol	No. of H Bonds	Length of Amino Acid
S100B	1.6	16, 26	-	6900	167	92
GFAP	1.1	18, 28	4.2.1	82,245	873	432
UCHL1	1.2	19, 29	4.2.4	18,957	336	223
NF-L	1.4	-	4.2.2	-		
Tau	1.5	20, 30	4.2.3	23,856–24,239	456–462	397

## Appendix A

**Table A1:** Summary of Clinical Trials.

Reference	No. of Patients	Inclusion Criteria	Exclusion Criteria	TBI Severity	Time of Injury	Biomarkers
[62]	109	Military personnel and veterans	Psychosis; schizophrenia; schizoaffective disorder; bipolar disorder; contraindication to MRI scanning	All	Median 5 years	Serum tau, NFL, and amyloid-beta 40 and 42
[63]	98	Military personnel deployed within the previous 18 months	Recent history of drug or alcohol abuse; current severe medical condition requiring long-term treatments; severe psychiatric conditions; severe neurological disorders	All	Most at least 18 months prior to the study	Plasma total tau
[68]	155	Patient in the ED of an adult level 1 trauma center; between 4 and 100 years old; had suspected head trauma requiring a head CT scan upon admission; available blood samples collected within 32 h of injury	Unidentified time of injury; history of head trauma 6 months prior to study; participating in another clinical study; active psychiatric, neurologic, and/or developmental disorders; admitted to the hospital’s special care unit; prisoners; persons in custody	All	0–8 h and 12–32 h	Serum GFAP, UCH-L1, and S100B
[80] NCT01132898	91	18 years old or older; speak and write English; diagnosed with non-penetrating TBI; enrolled in study within 1 year of injury	Pregnancy; contraindication to MRI; history of significant psychiatric or neurologic conditions	All	1 year	Extracellular vesicle GFAP and NFL
[81]	488	Service members and veterans; history of a TBI one year or more prior to study enrollment	Significant neurologic or psychiatric conditions	All	1 year or more	Serum total tau, GFAP, NFL, and UCH-L1
[82]	230	Subacute and chronic TBI patients; at least 18 years old; clinical diagnosis of nonpenetrating TBI; injury occurring within 1 year of enrollment	Contraindications to MRI; history of major neurologic or psychiatric conditions; pregnancy	All	30 days to 5 years	Serum NFL, GFAP, UCH-L1, and tau
[83]	343	Patients with mTBI recruited from the emergency departments of a level 1 trauma center and an out-patient clinic	Not fluent in Norwegian; pre-existing neurological, psychiatric, somatic, or substance use disorder; history of complicated TBI; presence of other major interfering trauma; presentation more than 40 h after injury	mTBI	Acute to 12 months	Plasma NFL, GFAP and tau
[84]	195	History of Operation Enduring Freedom/Operation Iraqi Freedom/Operation New Dawn deployment; history of combat exposure during any deployment; aged 18 years or older	History of moderate or severe TBI; history of major neurologic or psychiatric disorder	mTBI	Median 6.83–9.53 years	Exosomal and plasma NFL, TNF-alpha, IL-6, IL-10, and VEGF
[85] NCT01565551	107	External force trauma to the head; presentation to the ED of a participating trauma center; a clinically indicated brain CT scan within 24 h of injury	Pregnancy; comorbid life-threatening disease; incarceration; on psychiatric hold; non-English speaking	All	Less than 24 h	Plasma GFAP, UCH-L1, NFL, and total tau
[86]	21	18 years old or older; diagnosed with nonpenetrating moderate-to-severe TBI	Pregnancy; GCS equal to 3 associated with bilateral fixed and dilated pupils; normal head CT; interfering neurological comorbidities	moderate-severe TBI	up to 5 days	Serum exosomal GFAP, UCH-L1, NFL, and total tau
[87] NCT01565551	169	Presented to a participating level 1 trauma center within 24 h of injury; received a head CT scan upon admission to trauma center; 16 years old or older; able to provide informed consent	non-English speaking; pregnant; in custody; undergoing psychiatric evaluation; contraindications to MRI; pre-existing interfering medical or neurological conditions	mTBI	Less than 24 h	Plasma p-tau, total tau, and GFAP
[88]	584	Adult trauma patients that presented to the ED of a level 1 trauma center within 4 h of injury	younger than 18 years old; no history of trauma as their primary event; had known dementia, chronic psychosis, or active central nervous system pathology; pregnant; incarcerated; had a systolic blood pressure less than 100 mm Hg.	mild moderate TBI	less than 4 h to 180 h	Serum GFAP and UCH-L1
[89] NCT01990768	243	Head trauma patients presenting in the ED of one of 20 trauma centers; blunt or penetrating TBI; moderate-to-severe TBI; prehospital systolic blood pressure greater than 90 mm Hg; prehospital intravenous access; 15 years or older (or weight 50 kg or more if age unknown); estimated time lapse of less than 2 h between injury and hospital arrival	Prehospital GCS of 3 with no reactive pupil; estimated time from injury to start of study of more than 2 h; unknown time of injury; clinical suspicion of seizure activity; acute MI or stroke; known history of confounding medical conditions; CPR by EMS prior to randomization; burns more than 20% TBSA; prisoners; pregnancy; prehospital pro-coagulant drug given prior to randomization; opting out of the study	mod-sev TBI	0 to 24 h	Serum UCH-L1, GFAP, and MAP-2
